# A Novel Extracytoplasmic Function (ECF) Sigma Factor Regulates Virulence in *Pseudomonas aeruginosa*

**DOI:** 10.1371/journal.ppat.1000572

**Published:** 2009-09-04

**Authors:** María A. Llamas, Astrid van der Sar, Byron C. H. Chu, Marion Sparrius, Hans J. Vogel, Wilbert Bitter

**Affiliations:** 1 Department of Medical Microbiology, VU University Medical Center, Amsterdam, The Netherlands; 2 Structural Biology Research Group, Department of Biological Sciences, University of Calgary, Calgary, Alberta, Canada; Massachusetts General Hospital, United States of America

## Abstract

Next to the two-component and quorum sensing systems, cell-surface signaling (CSS) has been recently identified as an important regulatory system in *Pseudomonas aeruginosa*. CSS systems sense signals from outside the cell and transmit them into the cytoplasm. They generally consist of a TonB-dependent outer membrane receptor, a sigma factor regulator (or anti-sigma factor) in the cytoplasmic membrane, and an extracytoplasmic function (ECF) sigma factor. Upon perception of the extracellular signal by the receptor the ECF sigma factor is activated and promotes the transcription of a specific set of gene(s). Although most *P. aeruginosa* CSS systems are involved in the regulation of iron uptake, we have identified a novel system involved in the regulation of virulence. This CSS system, which has been designated PUMA3, has a number of unusual characteristics. The most obvious difference is the receptor component which is considerably smaller than that of other CSS outer membrane receptors and lacks a β-barrel domain. Homology modeling of PA0674 shows that this receptor is predicted to be a bilobal protein, with an N-terminal domain that resembles the N-terminal periplasmic signaling domain of CSS receptors, and a C-terminal domain that resembles the periplasmic C-terminal domains of the TolA/TonB proteins. Furthermore, the sigma factor regulator both inhibits the function of the ECF sigma factor and is required for its activity. By microarray analysis we show that PUMA3 regulates the expression of a number of genes encoding potential virulence factors, including a two-partner secretion (TPS) system. Using zebrafish (*Danio rerio*) embryos as a host we have demonstrated that the *P. aeruginosa* PUMA3-induced strain is more virulent than the wild-type. PUMA3 represents the first CSS system dedicated to the transcriptional activation of virulence functions in a human pathogen.

## Author Summary

*Pseudomonas aeruginosa* is a versatile pathogen; these bacteria are able to cause an infection in humans and other mammals, zebrafish, insects, nematodes and even plants. *P. aeruginosa* evolved an impressive amount of gene regulation systems to be able to express the right virulence genes under the right circumstances. The best studied examples of these are the two-component systems and the autoinducers. In addition, *P. aeruginosa* is also able to regulate virulence genes using the pyoverdine cell-surface signaling system (CSS). Genome analysis shows that there are multiple putative CSS systems in *P. aeruginosa*. In this paper we have studied a novel CSS system with a number of remarkable characteristics and show that this system is involved in the regulation of several putative virulence factors. Induction of this system leads to increased virulence in our zebrafish embryo infection model. Our study provides new insights into the regulation of virulence by *P. aeruginosa*.

## Introduction

The human opportunistic pathogen *Pseudomonas aeruginosa* is known for a high proportion of regulatory genes in its genome [1]. This is not only due to the number of two-component regulatory systems, but this bacterium also contain a large number of different cell-surface signaling (CSS) systems [2],[3]. CSS is a regulatory mechanism used by bacteria to sense signals from the extracellular medium and transmit them into the cytoplasm. CSS systems are generally composed of three different components, an alternative σ^70^ factor of the extracytoplasmic function (ECF) family, a sigma factor regulator located in the cytoplasmic membrane and an outer membrane receptor [2],[4],[5]. Sigma factors are essential subunits of prokaryotic RNA polymerase, they are involved in promoter recognition and transcription initiation. The primary sigma factor (RpoD), which is responsible for the majority of mRNA synthesis in exponentially growing cells, belongs to the σ^70^ family. This family also includes many alternative sigma factors that are nonessential proteins required only under certain circumstances [6],[7]. The largest and most diverged group within this family is the one including the ECF subfamily of sigma factors. ECF sigma factors are specially abundant in *P. aeruginosa* [8].

The outer membrane receptor of CSS systems is usually a member of the TonB-dependent receptor family. These receptors are mostly involved in the transport of iron-siderophore complexes across the outer membrane. To accomplish this task these receptors need to be energized by a protein complex in the cytoplasmic membrane. This protein complex is composed of TonB, ExbB and ExbD, of which the TonB protein is the one that actually makes contact with the outer membrane receptor, hence the name TonB-dependent receptors [9],[10]. TonB interacts with a specific region of the TonB-dependent receptors, generally known as the TonB box [11]. Coupling with the cytoplasmic membrane is necessary because the iron-siderophore complex has to be actively transported across the outer membrane, where there is no source of energy available. All TonB-dependent receptors possess the same structural components: a 22 antiparallel stranded β-barrel, an N-terminal globular domain known as the cork or plug domain that occludes the opening of the β-barrel and a TonB box that extends into the periplasm [10]. However, not all TonB-dependent receptors are involved in CSS, only a subfamily known as TonB-dependent transducers [12]. This subfamily can be easily distinguished from other TonB-dependent receptors on the basis of an N-terminal extension of approximately 70–80 amino acids [13]. This extension determines the specificity of the transduction pathway, but has no effect on the binding and transport of the siderophore [14]. This domain is thought to interact with the sigma factor regulator, which is located in the cytoplasmic membrane.

For *P. aeruginosa*'s own siderophore pyoverdine the signal transduction pathway of CSS starts with binding of the inducing signal Fe-pyoverdine to its outer membrane receptor FpvA, which results in the activation of two ECF sigma factors, PvdS and FpvI. Upon activation, PvdS binds the RNA polymerase core enzyme and directs it to the promoter upstream of the genes required for pyoverdine production and also of the genes encoding the virulence factors exotoxin A and PrpL [15]. Activated FpvI bound to the RNA polymerase initiates transcription of *fpvA* [16].

In addition to FpvI and PvdS, *P. aeruginosa* contains another twelve iron starvation sigma factors [17] that are probably part of a CSS pathway [2],[3]. Most of these *P. aeruginosa* iron starvation sigma factors control iron uptake via haem, via citrate or via heterologous siderophores, such as ferrichrome, ferrioxamine B and mycobactin [3], [18]–[20]. There are also two *P. aeruginosa* iron-starvation sigma factors that seem to regulate the uptake of a metal ion(s) different than iron, probably zinc or manganese [3]. The last *P. aeruginosa* iron starvation sigma factor is the one encoded by the PA0675 gene (named *pigD* in the Pseudomonas Genome Project database). This gene is clustered with a gene encoding a putative sigma factor regulator (PA0676 or *pigE*) and with one encoding a putative receptor (PA0674 or *pigC*). *In silico* analysis of this CSS system, which has been designated PUMA3, showed that it has a number of specific and unusual characteristics. The most obvious difference is the receptor component. The PA0674 receptor is considerably smaller (23 KDa) than that of other CSS outer membrane receptors (75–85 KDa). It contains the N-terminal extension typical of TonB-dependent receptors involved in signaling (Figure S1, Supporting Information), but does not have the C-terminal β-barrel domain typical of these receptors. Moreover, PA0674 seems to form a single operon with the ECF-encoding gene PA0675, while the sigma factor regulator gene PA0676 seems to form a different transcriptional unit. This is in contrast to all other CSS systems in which the genes encoding the sigma factor and the sigma factor regulator are forming an operon [3]. Interestingly, the synthesis of the PA0674 receptor is induced upon interaction of *P. aeruginosa* with human airway epithelial cells [21],[22], which suggests that this CSS system could be active *in vivo*.

This work was aimed at characterizing this novel *P. aeruginosa* CSS system. To get more information about its unusual receptor component, a homology model for the PA0674 protein has been constructed. The PUMA3 target genes were identified by microarray analysis of cells overexpressing the PA0675 ECF sigma factor. These analyses show that this CSS system is involved in the regulation of at least 27 genes, including genes encoding secreted proteins and components of secretion systems. Although the role of most of these regulated genes has not been established yet, we have demonstrated, using zebrafish (*Danio rerio*) embryos as an infection model, that PUMA3 is involved in the regulation of *P. aeruginosa* virulence. Therefore, we propose to rename the components of this system VreA (PA0674), VreI (PA0675) and VreR (PA0676) (from virulence regulator involving ECF sigma factor).

## Results

### VreA (PA0674) domain identification and homology modeling

Bioinformatic analysis predicts that the VreA receptor contains a signal sequence (SS) of 25 amino acids and separate amino-(N-) and carboxy-(C)-terminal domains (NTD and CTD, respectively) (Figure S2, Supporting Information). The predicted mature domain of VreA was submitted to several secondary structure prediction servers, including PSI-PRED [23]. The consensus results indicate that the NTD consists of residues 29–115 and the CTD of residues 133–238, which are separated by a short linker (residues 116–132).

**Figure 1:**
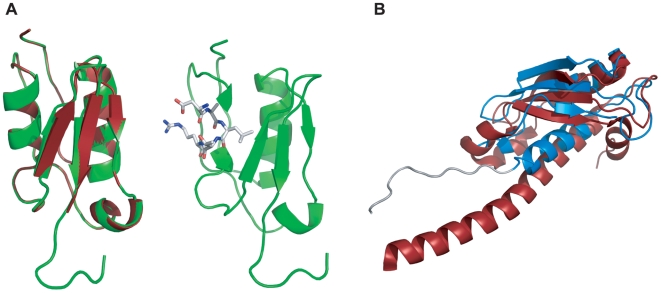
Homology structural model of VreA (PA0674). (A) VreA/NTD homology model. On the left is shown the structure of VreA/NTD (green) superimposed on the periplasmic signaling domain structure of FpvA (shown in red; PDB ID:2O5P). The two structures superimpose with a backbone Cα RMSD of 0.09 Å. On the right is shown the VreA/NTD structure with the predicted TonB-box residues (88-DALTR-92) drawn as sticks. (B) VreA/CTD homology model. The structure of the VreA/CTD (blue) superimposed on the structure of the TolA C-terminal domain from *P. aeruginosa* (shown in red; PDB 1D: 1LRO). The backbone Cα RMSD is 7.04 Å.

BLASTp results of the full-length VreA sequence against the Protein Databank revealed that residues 60–115 have a strong structural homology to the Secretin and TonB N-terminus (STN) domain superfamily. The highest ranked homologous structure (30% sequence identity) corresponds to the periplasmic signaling domain (residues 1–117) of the *P. aeruginosa* ferripyoverdine receptor FpvA. Since the structure of this protein is known, FpvA was used as a template for model building using a structure-based sequence alignment. Superimposition of residues 39–120 of the VreA/NTD structure to residues 1–82 of the signaling domain structure of FpvA reveals that the two structures are nearly identical, with a backbone Cα RMSD of 0.09 Å (Figure 1A). The structure of the VreA/NTD displays a β-α-β fold, with two α-helices positioned side-by-side and sandwiched between two-stranded and three-stranded β-sheets. Despite the high degree of homology between the model and template structures, an interesting difference arises with respect to the location of the expected TonB-box of VreA. In the structure of FpvA, the signaling domain is located before the TonB-box [24], while in the VreA/NTD structure the predicted TonB-box (88-DALTR-92) is found in α-helix 3 of the signaling domain (Figure 1A).

Submission of the C-terminal domain (CTD) sequence to the SUPERFAMILY server [25] revealed a strong structural homology to the C-terminal domain of the TolA/TonB protein superfamily, which was not detected by BLASTp or the Protein Model Portal [26]. The SUPERFAMILY server method is optimized to find homologues of protein sequences with low sequence identity. The crystal structure of the C-terminal periplasmic domain of *P. aeruginosa* TolA protein was identified as the closest structural domain despite a low overall sequence identity of 9.3%. TolA is part of the Tol-Pal (Tol-OprL in *Pseudomonas*) membrane complex, which is mainly involved in maintaining the integrity of the outer membrane [27],[28]. TolA is structurally and functionally related to the TonB protein, both of which belong to the TolA/TonB protein superfamily. The final homology model of VreA/CTD encompasses residues 124–233 and includes a portion of the short linker region. Structural alignment of the C-terminal domains of VreA and TolA reveals a backbone Cα RMSD of 7.04 Å (Figure 1B). The VreA and TolA C-terminal domains both adopt the same central secondary structure fold, β_(2)_-α-β, in which the three-stranded β-sheet is packed against two α-helices. The VreA/CTD homology model differs primarily from the TolA/CTD with respect to its shorter α-helix 1 and β-strand 3, which could have functional implications since both are directly involved in the interaction of TolA with other proteins [29].

These predictions would indicate that this putative VreA receptor is not located in the outer membrane, but in the periplasm. To study this in more detail we generated an influenza hemagglutinin (HA) epitope-tagged version of VreA and expressed this chimeric gene at low levels in the wild-type strain and in the PA0676 mutant, which does not produce the putative inner membrane regulator VreR. Although VreA is partially membrane associated, the majority is soluble (Figure S3) and therefore probably located in the periplasm. Furthermore, the presence or absence of the inner membrane regulator VreR did not affect stability and localization of VreA. Remarkably, the apparent molecular weight of VreA was higher than expected (*i.e.* 34 kD in stead of 23), which means that VreA is posttranslationally modified or has a secondary structure that affects migration in SDS-PAGE.

### Genes regulated by the *P. aeruginosa* ECF sigma factor VreI (PA0675)

**Figure 2:**
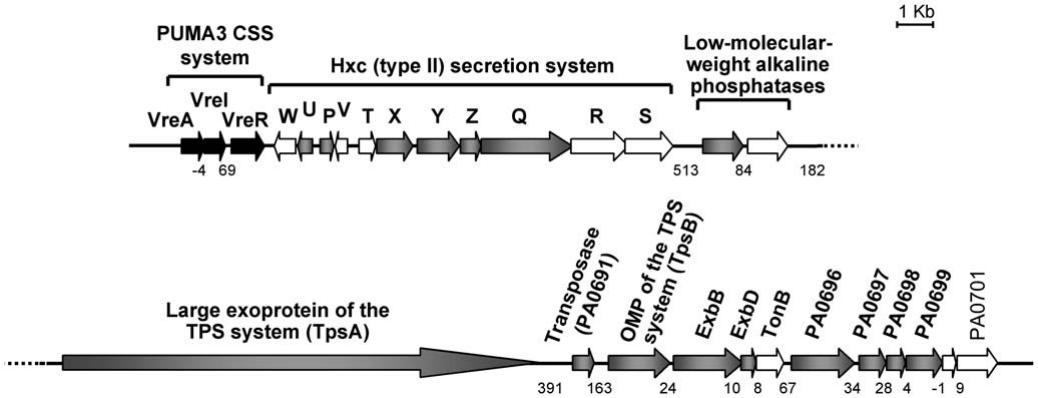
Genetic organization of the PUMA3 CSS system (black arrows) and part of the VreI (PA0675) regulon (grey arrows). Induction was determined by microarray analysis (Table 1). The arrows represent the different genes and their transcriptional orientation. Above each gene, the name of the encoded protein or the PA number (http://www.pseudomonas.com) is indicated. Numbers below the map indicate the distance (in base pairs) between adjacent genes; negative numbers indicate that the genes overlap the indicated number of base pairs. CSS, cell-surface signaling; TPS, two-partner secretion; OMP, outer membrane protein.

To identify genes whose transcription might be regulated by the VreI ECF sigma factor, total RNA from *P. aeruginosa* cells overexpressing the *vreI* gene from the pMUM3 plasmid was isolated and subjected to cDNA microarray analysis. Overexpression of ECF sigma factors usually results in the expression of the sigma-dependent genes in the absence of the inducing signal [3],[14],[16],[19]. As listed in Table 1, overexpression of *vreI* upregulates 30 genes (including the *vreI* gene itself that was overexpressed, and *vreA* and *vreR* that were also partially present on the pMUM3 plasmid and therefore overexpressed). Most regulated genes are located immediately downstream to the PUMA3 locus (Figure 2), as is often the case of genes regulated by ECF sigma factors. These genes encode: components of the Hxc type II secretion system (PA0677-PA0687) involved in the secretion of alkaline phosphatase (PA0688) [30], a putative two-partner secretion system (TPS) (PA0690 and PA0692), a putative transposase (PA0691), *exbBD* homologues (PA0693 and PA0694), three hypothetical proteins (PA0696, PA0697, and PA0698) two of them containing predicted signal peptides, and a putative peptididyl-prolyl cis-trans isomerase (PA0699) (Table 1). The putative secreted protein of the two partner secretion system (PA0690) belongs to a family of high-molecular-weight surface-exposed proteins involved in cell adhesion and pathogen dissemination [31],[32]. In addition, VreI seems to control the expression of a small number of other genes located in different loci of the *P. aeruginosa* genome. These include genes encoding an ECF sigma factor (PA0149), a hypothetical protein (PA0532), three putative cytoplasmic membrane proteins (PA1652, PA2404 and PA2784), two putative ATPases of ABC-transport systems (PA0716 and PA4192), two putative lipoproteins (PA2349 and PA5405), a homologue to the Fur regulator (PA2384), and a putative transcriptional regulator (PA5403).

**Table 1:** Genes of *P. aeruginosa* PAO1 with increased expression in cells overexpressing the *vreI* ECF sigma factor.

ORF^a^	Gene	Function or class^b^	Fold change VreI vs WT
PA0149		ECF sigma factor	7.4
PA0532		HUU	16.8
**PA0674**	***vreA***	**N-terminal half similar to TonB-dependent transducers**	**10.3**
**PA0675**	***vreI***	**ECF sigma factor**	**>100**
**PA0676**	***vreR***	**Transmembrane sensor (sigma factor regulator)**	**>100**
**PA0678**	***hxcU***	**Type II secretion system protein**	**5.6**
**PA0679**	***hxcP***	**Type II secretion system protein**	**9.3**
**PA0682**	***hxcX***	**Type II secretion system protein**	**8.5**
**PA0683**	***hxcY***	**Type II secretion system protein**	**18.6**
**PA0684**	***hxcZ***	**Type II secretion system protein**	**3.4**
**PA0685**	***hxcQ***	**Type II secretion system protein**	**4.4**
**PA0688**		**Low molecular weight alkaline phosphatase (PhoA)**	**5.2**
**PA0690**	***tpsA***	**Large exoprotein secreted by the TPS pathway**	**7.9**
**PA0691**		**Similar to transposase**	**16.2**
**PA0692**	***tpsB***	**Outer membrane transporter of the TPS pathway**	**17.8**
**PA0693**	***exbB2***	**Cytoplasmic membrane protein ExbB2**	**5.0**
**PA0694**	***exbD2***	**Cytoplasmic membrane protein ExbD2**	**3.2**
**PA0696**		**HUU; predicted signal peptide**	**17.0**
**PA0697**		**HUU; predicted signal peptide and a DNA binding motif**	**25.9**
**PA0698**		**HUU**	**3.2**
**PA0699**		**Peptidyl-prolyl cis-trans isomerase, PpiC-type**	**2.6**
**PA0716**		**Putative ATP-binding component of ABC transport system**	**3.9**
PA1652		HUU; membrane protein	4.9
PA2349		54% similar to putative lipoprotein YaeC of *E. coli*	3.1
PA2384		Probable Fur, Fe^2+^/Zn^2+^ uptake regulation protein	3.1
PA2404		HUU; membrane protein	3.0
PA2784		HUU; membrane protein	2.5
PA4192		Probable ATP-binding component of polar amino acid ABC transport system	9.7
PA5403		Probable transcriptional regulator	10.3
PA5405		HUU; Putative lipoprotein export signal (predicted by LipoP)	5.8

a PA number attributed in the *P. aeruginosa* genome annotation project (http://www.pseudomonas.com) [52].

b The functions of the encoded proteins are indicated according to the PAO1 genome annotation [52]. The genes located immediately downstream to the PUMA3 CSS locus are shown in bold. ECF, extracytoplasmic function; HUU, hypothetical, unclassified, unknown; TPS, two partner secretion.

**Figure 3:**
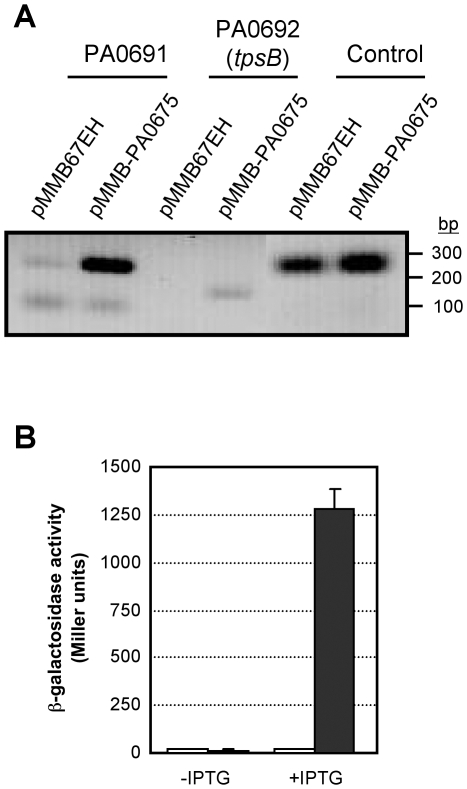
Validation of the microarray analysis by RT-PCR and B-galactosidase assay. (A) Gel electrophoresis of the cDNA amplified with primers within the PA0691, PA0692 and the control gene PA0636 from total RNA of *P. aeruginosa* cells carrying the plasmids pMMB67EH (empty plasmid) or pMUM3 (overexpressing the *vreI* ECF sigma factor). Positions of molecular size markers (in base pairs) are indicated. Negative controls containing the same amounts of RNA, primers and inactivated reverse transcriptase, were included in this assay (not shown). (B) *P. aeruginosa* PAO1 (wild-type) cells containing the *lacZ* transcriptional fusion pMP0691, and the plasmids pMMB67EH (empty plasmid) (white bars) or pMUM3 (grey bars), were grown in LB with or without 1 mM IPTG. β-galactosidase activity was then measured as described in Materials and Methods.

To validate the microarray results, the expression of some VreI-regulated genes was analyzed by RT-PCR. Primers within two VreI-regulated genes, PA0691 and PA0692, were designed to determine the mRNA levels in *P. aeruginosa* cells overexpressing the VreI ECF sigma factor. As shown in Figure 3A, the expression of both genes was induced by VreI, but not the expression of the control gene PA0636. VreI-mediated induction of PA0691 was also confirmed using a transcriptional fusion of the PA0691 promoter region to *lacZ*. Overexpression of the sigma factor *vreI* leads to a 25-fold increase in the PA0691 promoter activity when cells are cultured in presence of 1 mM IPTG (Figure 3B). Since *vreI* is under control of the P*tac* promoter, this inducing condition is expected to result in an increased expression of the PA0675-regulated genes.

### *In vivo* expression of PUMA3-regulated genes

**Figure 4:**
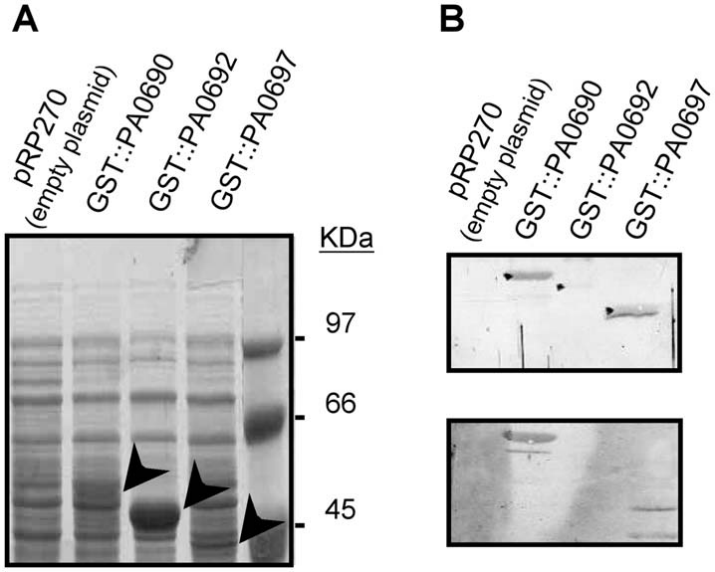
Presence of antibodies against VreI (PA0675)-regulated proteins in the serum of *P. aeruginosa* infected patients. (A) Expression of GST-tagged proteins in *E. coli*. *E. coli* DH5 α cells bearing the indicated GST fusion were grown overnight in LB liquid medium and samples were harvested for total protein preparation as described in Materials and Methods. About 10^8^ cells were loaded on each lane and proteins were separated by 12.5% (w/v) polyacrylamide SDS-PAGE and Coomassie blue stained. Arrows indicate the position of the GST-tagged proteins. The position of the molecular size marker is indicated on the right. (B) Immunodetection of purified GST-tagged proteins using the serum of two different CF patients infected with *P. aeruginosa*. Western blot reactions were revealed by use of the peroxidase colorimetric method [55].

Previous experiments have shown that the PUMA3 CSS system appears to be induced *in vivo*, since interaction of *P. aeruginosa* with human airway epithelial cells induces the expression of many VreI-regulated genes (Tables S1 and S2, Supporting Information) [21],[22]. In order to determine whether VreI-regulated genes are synthesized *in vivo*, we analyzed the presence of antibodies against VreI-regulated proteins in the serum of *P. aeruginosa* infected patients. To this end, predicted highly antigenic internal fragments of the PA0690 (TpsA), PA0692 (TpsB) and PA0697 genes were fused to a glutathione S-transferase (GST) gene and overproduced in *E. coli* (Figure 4A). The fusion proteins were then purified using Glutathione Sepharose 4B columns. Subsequently, these purified chimera proteins were used to detect the presence of antibodies in the serum of *P. aeruginosa* infected patients. We tested in total the serum of 25 different patients, 7 with positive blood culture for *P. aeruginosa* and 18 cystic fibrosis (CF) patients. Antibodies against the secreted component of the TPS system, the PA0690/TpsA protein, were present in the serum of 5 of the 7 patients with positive blood culture for *P. aeruginosa* (71.4%) and in the serum of 12 of the 18 CF patients tested (66.7%) (Figure 4B). However, antibodies against the second component of the TPS system, the outer membrane transporter PA0692/TpsB could not be detected with any of these sera (Figure 4B). The third protein tested, PA0697, which contains a putative signal sequence, was detected with 4 (57.1%) of the sera from patients with positive blood culture for *P. aeruginosa* and with 10 (55.5%) of the CF patients sera (Figure 4B). The presence of antibodies against these proteins indicates that they are being expressed during infection. Since mRNA levels of these genes are extremely low under non-inducing conditions, this result also suggests that the PUMA3 CSS system is induced in these patients.

### Analysis of VreI activity and stability in a VreR (PA0676) sigma factor regulator mutant

**Figure 5:**
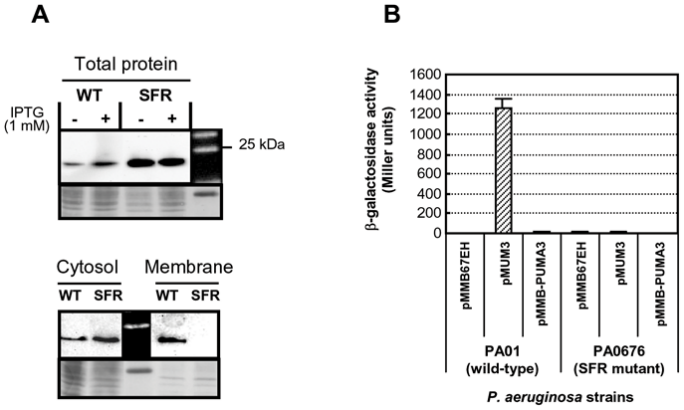
Analysis of VreI (PA0675) stability and activity. (A) SDS-PAGE of *P. aeruginosa* wild-type cells (WT) and the *vreR* sigma factor regulator mutant bearing the pMUM3RσHA-tag plasmid coding for the VreI-HA-tagged protein. Total proteins (upper panel), and cytosol and membrane fractions (lower panel) were separated in 15% (w/v) acrylamide gel. Log phase cells were incubated 45–60 min with (+ in upper panel, and all samples in lower panel) or without (− in upper panel) 1 mM IPTG. (B) β-galactosidase activity of *P. aeruginosa* wild-type or PA0676 mutant cells containing the pMP0691bKm plasmid (PA0691::*lacZ* transcriptional fusion) and the pMMB67EH (empty), the pMUM3 (overexpressing the *vreI* ECF sigma factor) or the pMMB-PUMA3 (overexpressing the whole PUMA3 CSS system) plasmid. Cells were grown overnight in LB liquid medium in the presence of 1 mM IPTG. The β-galactosidase activity is expressed in Miller Units.

In order to determine the role of the sigma factor regulator VreR in the PUMA3 signaling pathway, we analyzed the stability and activity of the VreI ECF sigma factor in a *vreR* mutant. To analyze the stability of this sigma factor, we first constructed the pMUM3RσHA-tag plasmid in which the *vreI* gene is C-terminal tagged with the HA epitope. This plasmid and the control plasmid pMUM3 were then transferred to the *P. aeruginosa* PAO1 wild-type (WT) strain and the *vreR* mutant (sigma factor regulator mutant). The presence of the VreI-HAtag protein was analyzed by Western blot using an anti-HAtag antibody. As shown in Figure 5A, the VreI-HAtag protein (22 kDa) could be detected in strains bearing the pMUM3RσHA-tag plasmid, whereas it could not be detected in strains bearing the control plasmid (data not shown). The addition of 1 mM IPTG slightly increases VreI-HAtag production, which is under control of the P*tac* promoter (Figure 5A, upper panel). Interestingly, the VreI ECF sigma factor seems to be more stable in absence of the sigma factor regulator as the amount of this protein in the *vreR* mutant is considerably higher that in the wild-type strain (Figure 5A). Analysis of the cytosol and membrane fractions of both strains showed that VreI is associated to the membrane through the VreR sigma factor regulator since the VreI-HAtag protein could not be detected in the membrane fraction in absence of this protein (Figure 5A, lower panel). Although there is more VreI sigma factor in absence of VreR, this sigma factor is not active in this condition (Figure 5B), since overexpression of *vreI* in the *vreR* mutant does not increase PA0691 promoter activity, while it does in the wild-type strain (Figure 5B). Overexpression of the whole PUMA3 system (receptor, ECF sigma factor and sigma factor regulator) from the pMMB-PUMA3 plasmid does not increase PA0691 promoter activity (Figure 5B), possibly due to the simultaneous overexpression of the *vreR* gene encoding the sigma factor regulator component. In conclusion, VreR is an anti-sigma regulator for VreI that is both required for the function of VreI and inhibits its activity under non-inducing conditions.

### Analysis of *P. aeruginosa* virulence in zebrafish (*Danio rerio*) embryos

Although the role of most *P. aeruginosa* PUMA3-induced genes has not been established yet, the fact that some of them encode secreted proteins and components of secretion systems suggests that the PUMA3 CSS system could be involved in regulation of virulence. For this reason we decided to analyze *P. aeruginosa* virulence. Therefore, we used a novel infection model for *P. aeruginosa* using zebrafish (*Danio rerio*) embryos as a host. The zebrafish model has a number of advantages over other models of infection [33]. One of them is that zebrafish embryos are transparent, which allows the analysis of bacterial infections *in situ*, in real time and at a high resolution by using fluorescent microorganisms. Recently, zebrafish embryos have been reported to be a suitable model for *P. aeruginosa* [34],[35].

**Figure 6:**
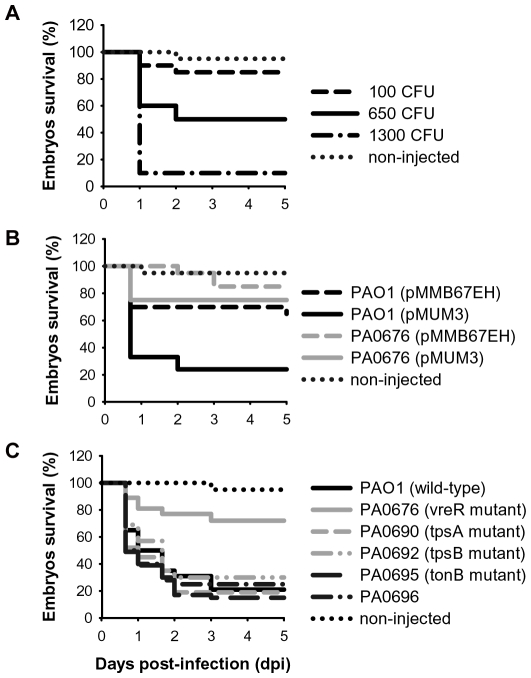
Klapan-Meier embryo survival curves following infection with *P. aeruginosa*. (A) Dose-dependent response in embryo survival to *P. aeruginosa* infection. Groups of 28–30 hpf embryos (n = 20 embryos/group) were inoculated with a low dose (100 CFU), an intermediate dose (650 CFU), or a high dose (1300 CFU) of PAO1 wild-type cells. Uninfected control is shown (non-injected). (B) Embryo survival following infection with ∼500–800 CFU of *P. aeruginosa* wild-type strain or with the PA0676 *vreR* sigma factor regulator mutant bearing the empty pMMB67EH plasmid or the pMUM3 plasmid in which the PUMA3 system is induced by overexpression of *vreI*. The data are representative of 3 replicates with 20 embryos/group in each replicate. (C) Embryo survival after infection with ∼700 CFU of *P. aeruginosa* wild-type strain or with the mutants PA0676 *vreR*, PA0690 *tpsA*, PA0692 *tpsB*, PA0695 *tonB*-homologue, and PA0696 gene bearing the pMUM3 plasmid overexpressing the *vreI* ECF sigma factor were microinjected in zebrafish embryos. The data are representative of 2 replicates with 20 embryos/group in each replicate.

In order to set up the model, we analyzed first whether *P. aeruginosa* could infect 28–30 hours-post-fertilization (hpf) embryos. To this end we introduced *P. aeruginosa* PAO1 wild-type strain into the zebrafish embryo by microinjection in the caudal vein. We observed that *P. aeruginosa* was able to lethally infect the embryos in a dose dependent manner (Figure 6A). Embryos were resistant to low doses of bacteria (150–200 colony forming units, CFU), but increased mortality was observed with larger inocula (∼400–1300 CFU) (Figure 6A). These experiments also showed that *P. aeruginosa* kills the embryos within the first two days-post-infection (dpi); embryos that were alive after this time usually were able to clear the *P. aeruginosa* infection and developed normally.

**Figure 7:**
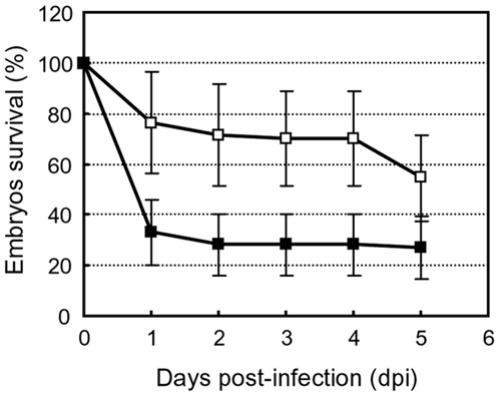
Effect of PUMA3 induction on *P. aeruginosa* virulence. Embryo survival following infection with ∼360–750 CFU of *P. aeruginosa* wild-type strain bearing the pMMB67EH empty plasmid (open squares) or the pMUM3 plasmid (close squares) that induces PUMA3 by overexpression of the *vreI* ECF sigma factor. Plotted data indicates the mean values of five independent experiments with 20 embryos/group in each replicate; whiskers indicate standard errors of the mean.

Then, we analyzed the virulence of the *P. aeruginosa* PUMA3-induced strain, by overexpression of the *vreI* ECF sigma factor, in zebrafish embryos. As shown in Figure 7, infection with the *P. aeruginosa* PUMA3-induced strain resulted in a significant increase of zebrafish embryo mortality. This effect was repeatedly shown in 5 different experiments using groups of 20 embryos. To demonstrate that this effect is specific for PUMA3 induction, we also infected the embryos with the *vreR* sigma factor regulator mutant bearing the pMUM3 plasmid that overexpresses the VreI sigma factor. We have shown previously that overexpression of *vreI* in this mutant does not lead to upregulation of the PUMA3-controlled genes (Figure 5B). As expected, induction of PUMA3 in the *vreR* mutant did not result in an increase in *P. aeruginosa* virulence (Figure 6B), which confirms the direct involvement of VreI in the increased *P. aeruginosa* virulence.

We next assessed the role of different PUMA3-regulated genes in VreI-induced virulence. To this end, the pMUM3 plasmid was introduced in transposon insertion mutants of PUMA3-regulated genes encoding potential virulence factors, such as both components of the TPS system *tpsA* and *tpsB* (PA0690 and PA0692, respectively), the *tonB* homologue PA0695 and the putative secreted protein PA0696. Subsequently, these different mutants were injected in zebrafish embryos. Unfortunately, all mutants, except the *vreR* sigma factor regulator mutant described previously, were as virulent as the wild-type strain (Figure 6C). This means that none of these potential virulent factors is by itself responsible of the VreI-induced lethality in zebrafish embryos. Possibly a combination of these factors is responsible for this phenotype, or some of the other VreI-regulated genes.

### Localization of *P. aeruginosa* within zebrafish embryos

**Figure 8:**
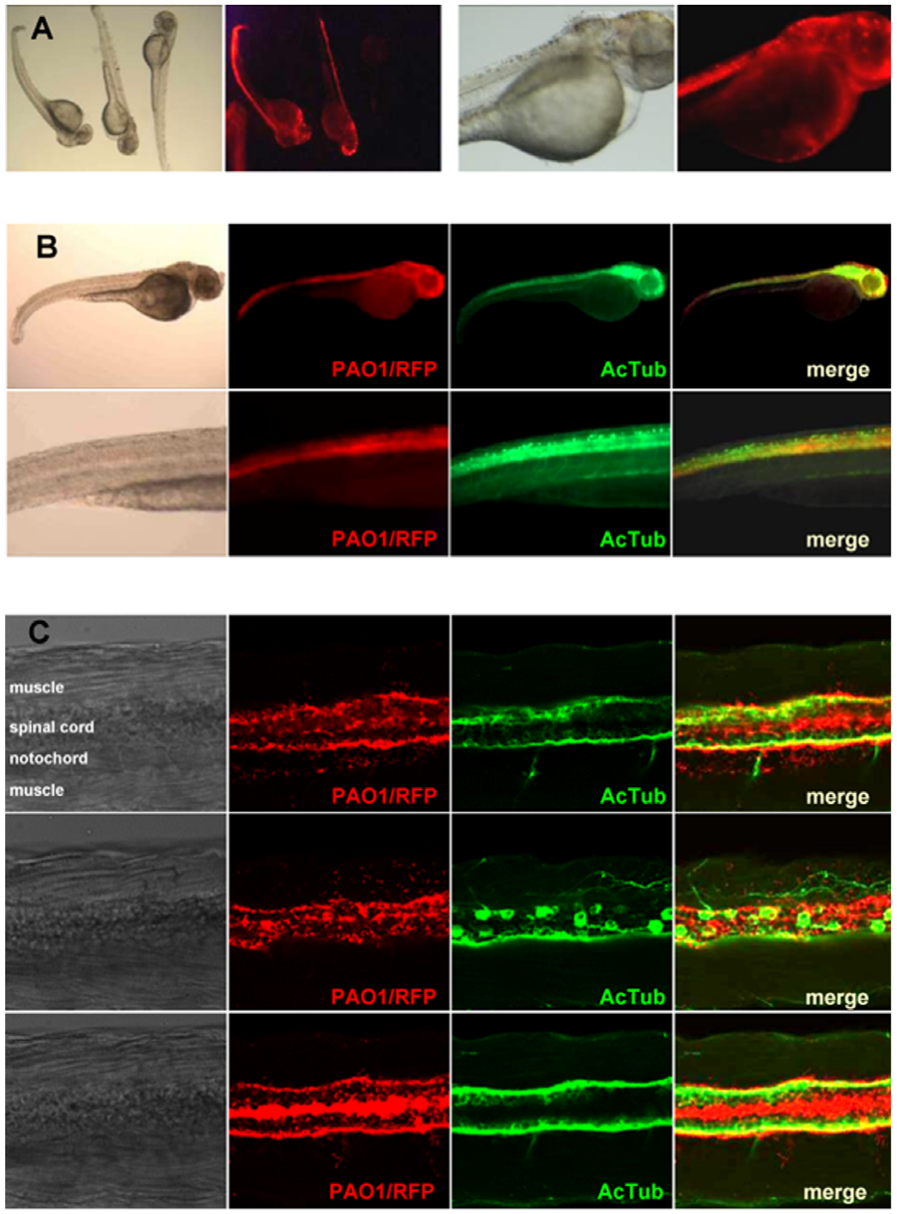
Localization of *P. aeruginosa* within zebrafish embryos. (A) Fluorescent images of embryos at 1 dpi with an intermediate dose of *P. aeruginosa* PAO1/RFP cells overexpressing *vreI* from the pMUM3 plasmid. These embryos were highly infected and normally died by 24–30 hpi. Embryos in whose PAO1/RFP was not visible at 1 dpi (*i.e.* embryo at the right side of the first panel) survive by clearing the infection and were indistinguishable from the non-injected group. (B) Fluorescent images of embryos infected with PAO1/RFP (red channel) and subjected to whole mount immunohistochemistry using an anti-acetylated tubulin (AcTub) monoclonal antibody that specifically recognizes the nerves of the embryo (green channel). The last panel shows the red/green overlay. (C) Confocal images of three different focal planes of the embryo shown in (B) with similar color coding. All PAO1/RFP panels clearly show the concentration of *P. aeruginosa* in and around the spinal cord. In addition single bacteria can be seen in the muscle tissue. No colocalization of neuronal cell bodies or axons with PAO1/RFP was seen. However, a close contact between the axon tracts in the spinal cord and the PAO1/RFP is observed.

Zebrafish embryos infected with red fluorescent *P. aeruginosa* (PAO1/RFP) bearing the pMMB67EH (empty plasmid) or the pMUM3 (overexpressing *vreI*) plasmid were microscopically observed to follow the progression of the infection (Figure 8A). In the first hours after infection, fluorescence was undetectable (data not shown), but with the progression of the infection, fluorescent bacteria were clearly detectable in the embryos at 20–24 hpi (Figure 8A). The amount of fluorescence in the embryos correlated with a slower blood circulation and decreased heartbeat when compared with healthy embryos (in which fluorescence was undetectable), and also with severe damages of the tissues, mainly in the tail (Figure 8A). Affected embryos presented increasing red fluorescence and normally died within the first 24–30 hpi, whereas infected but apparently healthy embryos were able to survive and develop normally. There were no obvious differences between the phenotype of moribund embryos infected with the PUMA3-induced strain or with the non-induced strains (data not shown). Both strains produce a similar necrotic cell death that starts in the tail and extends to other tissues. The difference between both strains lies in the fact that a considerably higher percentage of embryos infected with the induced strain died due to the proliferation of the *P. aeruginosa* infection (Figure 6B and 8A).

The microscopy studies also showed that *P. aeruginosa*, which was microinjected in the blood stream, was able to extravasate and infect other tissues, mainly what appears to be brain and spinal cord of the embryos (Figure 8A). By whole mount immunohistochemistry of embryos infected with PAO1/RFP using an antibody that specifically recognizes Acetylated tubulin (AcTub) present in neurons and axons of the embryo, we observed co-localization of bacteria with fluorescent brain and spinal cord tissues (Figure 8B). More in depth analysis with confocal microscopy clearly showed many bacteria in the center and around the nerve bundles of the spinal cord (Figure 8C). No colocalization with axons or neuronal cell bodies was observed (Figure 8C, middle panel), which suggests that *P. aeruginosa* bacteria reside in non-neuronal cells or extracellularly. In addition single bacteria were observed in the muscles (Figure 8C, middle panel).

## Discussion

In this work, we report the identification and characterization of a novel CSS system, designated PUMA3, which has a number of specific and unusual characteristics, and is involved in the regulation of *P. aeruginosa* virulence. The most obvious difference of PUMA3 with all other CSS systems is the receptor component VreA. The structural modeling of VreA predicts a bilobal protein (Figure S2), with an N-terminal domain (NTD) that resembles the N-terminal periplasmic signaling domain of TonB-dependent transducers, such as FpvA, and a C-terminal domain (CTD) that resembles the periplasmic C-terminal domain of the TolA/TonB protein superfamily. The signaling domain of TonB-dependent transducers is the domain that interacts with the sigma factor regulator [36]. Therefore, it is likely that VreA/NTD interacts with the sigma factor regulator VreR, as illustrated in Figure S4. Although the structures of VreA/NTD and the signaling domain of FpvA are nearly identical (Figure 1A), an interesting difference is the location of the expected TonB-box of VreA. In FpvA, the signaling domain is located N-terminal to its TonB-box. In the apo-FpvA structure, the TonB-box is buried between the signaling domain and the plug/barrel domains, and forms a β-strand that interacts with the signaling domain. Upon ferric-pyoverdine binding this β-strand is displaced and free to interact with TonB in a β-strand lock-exchange mechanism [24]. In the VreA/NTD structure, the predicted TonB-box is found in α-helix 3 of the signaling domain fold (Figure 1A). If the VreA TonB-box interacts with the TonB protein in a similar mixed four-stranded β-sheet fashion as other reported TonB-dependent proteins a significant conformational change would be required.

The VreA/CTD showed, despite the low sequence identity, strong structural homology to the C-terminal domain of the TolA protein (Figure 1B). The Tol-Pal (Tol-OprL) system is organized into two protein complexes: a cytoplasmic membrane complex that consists of the TolQ, TolR, and TolA proteins, and an outer membrane-associated complex composed of TolB and Pal. TolA plays a central role by providing a bridge between the cytoplasmic and outer membranes via its interaction with the Pal lipoprotein [37]. The Tol proteins are parasitized by filamentous (Ff) bacteriophages and group A colicins [38],[39]. The N-terminal domain of the Ff phage g3p protein and the translocation domains of colicins interact directly with TolA during the processes of import through the cell envelope [40]. The TolA protein has functional analogy with the TonB protein. Especially the interaction of the C-terminal domain of TolA and the Ff phage g3p protein is similar with that of the C-terminal domain of TonB and the TonB-box of TonB-dependent receptors [11],[41]. Since the C-terminal domain of VreA is similar to TolA/TonB, it is tempting to speculate that VreA/CTD could interact with other partner proteins in the outer membrane (Figure S4). Based on bioinformatics analysis, it is clear that the predicted domain architecture of VreA is unique and has yet to be reported. Significantly, both domains of VreA are predicted to resemble proteins that form essential interactions with partner proteins required for signal transduction and bacterial virulence.

By microarray analysis of *P. aeruginosa* cells overexpressing the PUMA3 ECF sigma factor VreI, we have identified the genes regulated by this novel CSS system. It was shown previously that overexpression of the sigma factors results in the specific induction of the sigma-dependent genes in the absence of the inducing signal [3],[14],[16],[19]. The microarray analysis shows that the PUMA3 system controls the expression of 27 genes (Table 1), most of which are located directly downstream of the PUMA3 locus (Figure 2). As observed previously for other ECF sigma factors [3], overexpression of *vreI* does not result in an unspecific response and does not affect house-keeping genes. This is probably due to the fact that the RNA polymerase has a higher affinity for the house-keeping sigma factor RpoD (σ^70^) than for alternative sigma factors [42].

The interaction network of the *P. aeruginosa* VreA receptor with other proteins using the STRING database [43] shows the physical and functional connectivity of this protein not only with the other two components of the PUMA3 system VreI and VreR, but also with most PUMA3-regulated proteins such as both components of the TPS system, ExbBD2, and the PA0696-PA0697-PA0699 proteins (Figure S5A, Supporting Information). PUMA3 homologues are found in the genome of both *Pseudomonas fluorescens* Pf5 and Pf01, *Pseudomonas entomophila*, *Burkholderia vietnamiensis*, *Rhodopseudomonas palustris* and *Janthinobacterium sp* (Figure S5B). Interestingly, in all these bacteria, the PUMA3 gene cluster is associated with homologues of the TPS secretion system, of the ExbBD system and of PA0696. *P. fluorescens* contains an Hxc-like type II secretion system immediately downstream of PUMA3 and also a *tpsA*-like gene. In *B. vietnamiensis*, homologues of some of the *hxc*-like genes are located upstream of PUMA3, interrupted by a *tpsA*-like gene. The second component of the TPS pathway is located downstream of PUMA3, associated with *exbBD* homologues. In *R. palustris* and *Janthinobacterium sp.*, the PUMA3 cluster is repeated four times. Three of these clusters are followed by a *tpsA* homologue, and the fourth one by a *tpsB*-like gene associated with *exbBD* and PA0696 homologues. This gene association further suggests a role for PUMA3 in the regulation of the genes, especially *tpsA*.

Although most CSS systems described to date control the expression of their cognate TonB-dependent transducers, this does not seem to be the case of the PUMA3 CSS system. Microarray analysis did show an increase in *vreA* mRNA levels in cells overexpressing the VreI sigma factor (Table 1). However, because part of the *vreA* gene is also partially present on the *vreI* overexpressing plasmid this does not mean that *vreA* is induced upon activation of PUMA3. Direct analysis of *vreA* expression using a *vreA*::*lacZ* transcriptional fusion showed no differences between cells overexpressing or non-overexpressing *vreI* (data not shown). This difference between PUMA3 and other CSS systems is probably related to the unusual genetic organization of the PUMA3 system. In contrast to all CSS systems in which the sigma and sigma factor regulator genes are arranged in an operon, the sigma factor gene of the PUMA3 system seems to form an operon with the receptor gene (these genes overlap 4 bp), while the sigma factor regulator seems to be part of a different transcriptional unit (Figure 2). In this situation, regulation of the VreA receptor expression by the VreI sigma factor would imply that VreI also induces its own expression, which is unusual for ECF sigma factors.

Another interesting characteristic of PUMA3 is the role of the sigma factor regulator VreR in the signaling pathway. The function of this integral cytoplasmic membrane protein is to couple the signal perceived by the TonB-dependent transducer to the ECF sigma factor in the cytoplasm. The large periplasmic C-terminal part interacts with the receptor in the outer membrane, whereas the short cytoplasmic tail binds the ECF sigma factor [44],[45]. Currently, there is no structural data available for any member of this protein family, and the molecular mechanism by which these proteins work is not completely understood. It is generally considered an anti-sigma factor, based on the fact that overexpression of the ECF sigma factor results in constitutive induction of the CSS system [3],[19]. In accordance with this, overexpression of the sigma factor regulator results in a strongly reduced induction upon the presence of the extracellular signal [16]. However, for the PUMA3 CSS system the sigma factor regulator is in fact essential for VreI sigma factor activity (Figure 5B). This is also the case for the sigma factor regulator FecR of the *E. coli* ferric-citrate CSS system [4]. Our experiments also show that the PUMA3 ECF sigma factor is more stable in the absence of the sigma factor regulator and relocates to the cytosol (Figure 5A). A similar situation has been described for the sigma factor PvdS and its regulator FpvR; overexpression of *fpvR* results in increased degradation of the ECF sigma factor PvdS and possibly also FpvI [45],[46] and PvdS relocates partially to the cytosol in absence of the inner membrane regulator [47]. This means that these sigma factor regulators not only retain the ECF sigma factors at the cytoplasmic membrane in an inactive form, but possibly also deliver these to a specific endoprotease. Future experiments are necessary to show which protease is involved and what the exact role of the PUMA3 sigma factor regulator.

The PUMA3 CSS system appears to be induced *in vivo*, since the serum of the majority of *P. aeruginosa*-infected patients, including patients with positive blood culture for *P. aeruginosa* and cystic fibrosis patients, contains antibodies directed against PUMA3-regulated proteins (*i.e.* TpsA and PA0697) (Figure 4B). This means that these proteins are synthesized *in vivo* during infection and that the PUMA3 system is probably induced. This further suggests its activation by a host signal, which is consistent with the previous reports showing that interaction of *P. aeruginosa* with human airway epithelial cells induces the expression of many PUMA3-regulated genes [21],[22] (Tables S1 and S2).

Although the role of most *P. aeruginosa* PUMA3-induced genes has not been established yet, we have demonstrated that this CSS system is involved in the regulation of *P. aeruginosa* virulence. Infection of zebrafish embryos with the *P. aeruginosa* PUMA3-induced strain by overexpression of the *vreI* sigma factor results in a significant increase of embryo mortality (Figure 6B). This effect is VreI specific since its overexpression in a mutant in the sigma factor regulator, which is necessary for VreI activity (Figure 5B), did not result in increased virulence (Figure 6B). The induced lethality was visible after the first day of infection as the embryos that were alive at this point usually were able to clear the infection. Zebrafish embryos have been recently reported to be a suitable model for *P. aeruginosa* [34],[35]. Known attenuated *P. aeruginosa* mutants, such as mutants in type III secretion or in quorum sensing, are less virulent in zebrafish embryos. Moreover, key host determinants, such as phagocytosis, play an important role in zebrafish embryos pathogenesis, and, as in humans, phagocyte depletion increases the susceptibility of the embryos to *P. aeruginosa* infection [34]. Neutrophils and macrophages rapidly phagocytosed and killed *P. aeruginosa*, but if the amount of cells injected exceeds the phagocytic capacity of the embryo bacteria survive and grow causing the death of the embryo [34]. The PUMA3 CSS system seems to play a role in the first hours of infection. Induction of PUMA3 may result in *P. aeruginosa* resistance to phagocytosis leading to a lower survival of the embryos. Alternatively, the PUMA3-induced strain may replicate faster in the embryo, although the growth rate of these strains in vitro showed no difference (data not shown). The identification of the upregulated factor(s) responsible of the PUMA3-induced virulence will be essential to understand the mechanisms by which this CSS system induces virulence.

Using fluorescence microscopy, we have shown that *P. aeruginosa* is able to extravasate and infect other tissues, mainly the brain and spinal cord of the embryos (Figure 8). This pattern of infection seems to be specific for *P. aeruginosa* and completely different to the one caused by, for example, *Salmonella typhimurium*, which replicates either inside macrophages or extracellularly but always within the vascular system, or *Mycobacterium marinum*, which is located in clustered macrophages [33],[48].

In summary, in this work we have identified and characterized a novel CSS system that triggers expression of virulence factors, probably in response to a host signal. A similar function for a CSS system has only been described in the plant pathogen *Ralstonia solanacearum*, which uses a CSS system to regulate virulence in response to a non-diffusible plant signal [49],[50]. However, whereas the *Ralstonia* CSS system has all the characteristics of a normal CSS system, PUMA3 shows a number of new and unusual characteristics, and represents the first CSS system dedicated to the activation of virulence factors in a human pathogen. Our work also shows that CSS systems can be used for a broader purpose than the regulation of iron uptake.

## Materials and Methods

### VreA (PA0674) homology modeling

A homology model of the N-terminal and C-terminal domains of VreA (VreA/NTD and VreA/CTD, respectively) was built using the SWISS-MODEL server [51]. The VreA/NTD homology model was constructed using a structure-based sequence alignment (SBSA) and the crystal structure of the *P. aeruginosa* ferripyoverdine receptor FpvA (PDB ID: 2O5P) as template. Other structural homologues of FpvA, including the periplasmic signaling domains from the *E. coli* ferric citrate receptor FecA (PDB IDs: 2D1U and 1ZZV) and *P. putida* pseudobactin 358 receptor PupA (PDB ID: 2A02) were included in the SBSA. For the VreA/CTD domain, the template for model building was the C-terminal domain of TolA from *P. aeruginosa* (PDB ID: 1LRO). Other structural homologues including the C-terminal domains of TolA (PDB ID: 1S62) and TonB (PDB ID: 1XX3) from *E. coli* were used to construct an SBSA upon which the C-terminal domain sequence of VreA was initially threaded using the program Deepview (version 4.0), prior to submission to the SWISS-MODEL server.

### Other computer-assisted analysis

Sequence analysis of the PAO1 genome was performed at http://www.pseudomonas.com [52]. Signal peptides were predicted using the SignalP 3.0 Server available at http://www.cbs.dtu.dk/services/SignalP/ [53]. The functional associations of PUMA3 were predicted using the STRING 8 database at http://string.embl.de/ [43].

### Bacterial strains, plasmids, culture media, and growth conditions

The bacterial strains and plasmids used are listed in Table 2. *P. aeruginosa* PAO1 wild-type strain and all the *P. aeruginosa* transposon insertion mutants used were from the comprehensive *P. aeruginosa* transposon mutant library at the University of Washington Genome Center [54]. The locations of the mutations were confirmed by PCR with primers flanking the insertion sites. The strain ID (unique identifier) is given on the table, and further information on these mutants can be found at http://www.genome.washington.edu/UWGC/Pseudomonas/index.cfm.

**Table 2:** Bacterial strains and plasmids used in this study.

Strain or plasmid	Relevant characteristics^a^	References
*E. coli*		
DH5α	*supE*44 Δ*lac*U169 (φ80 *lacZ*ΔM15) *hsdR*1 *recA*1 *endA*1 *gyrA*96 *thi1 relA*1	[61]
HB101	*supE*44 *hsdS*20 *recA*13 *ara*14 *proA*2 *lacY*1 *galK*2 *rpsL*20 *xyl*-5 *mtl*-1	[62]
*P. aeruginosa*		
PAO1	Wild-type	[54]
PA0676/*vreR*	PA0676::IS*phoA*/hah derivative of PAO1 (ID 42533); Tc^R^	[54]
PA0690/*tpsA*	PA0690::IS*lacZ*/hah derivative of PAO1 (ID 14608); Tc^R^	[54]
PA0692/*tpsB*	PA0692::IS*lacZ*/hah derivative of PAO1 (ID 10896); Tc^R^	[54]
PA0695/*tonB*	PA0695::IS*lacZ*/hah derivative of PAO1 (ID 10838); Tc^R^	[54]
PA0696	PA0696::IS*lacZ*/hah derivative of PAO1 (ID 8944); Tc^R^	[54]
Plasmids		
pBBR1MCS-5	Gm^R^; *oriT*RK2	[60]
pBSL99	Source of the Km cassette; Ap^R^, Km^R^	[63]
pMMB67EH	IncQ broad-host range plasmid, *lacI^q^*; Ap^R^	[64]
pMP220	IncP broad-host-range *lacZ* fusion vector; Tc^R^	[56]
pRK600	Helper plasmid, *ori*ColE1, *mob*RK2, *tra*RK2; Cm^R^	[57]
pRP270	GST (gluthathione-S-transferase) fusion vector, Ap^R^	[65]
pUC18	Cloning vector, *ori*ColE1, *rop* mutant, α-*lacZ*	[66]
pUCMA3	pUC18 carrying in SmaI a 2.2-kb PCR fragment containing the *P. aeruginosa* PAO1 PA0675-PA0676 genes; Ap^R^	This study
pBBR-PoprF	pBBR1MCS-5 carrying in XhoI-HindII a 300-bp PCR fragment containing the *P. aeruginosa* PAO1 *oprF* promoter region	This study
pMUM3	pMMB67EH carrying the 1.4-kb KpnI-NheI insert from pUCMA3 containing the PA0675 gene; Ap^R^	This study
pMUM3Rσ	pMMB67EH carrying in EcoRI-BamHI a 1.46-kb PCR fragment containing the *P. aeruginosa* PAO1 PA0674-PA0675 genes; Ap^R^	This study
pMMB-PUMA3	pMMB67EH carrying in EcoRI-BamHI a 2.6-kb PCR fragment containing the *P. aeruginosa* PAO1 PA0674-PA0675-PA0676 genes; Ap^R^	This study
pMUM3Rσno-stop	pMMB67EH carrying in EcoRI-XbaI a 1.45-kb PCR fragment containing the *P. aeruginosa* PAO1 PA0674-PA0675 genes, the last one without stop codon; Ap^R^	This study
pMUM3RσHA-tag	pMUM3Rσno-stop carrying the HA-tag epitope in the C-terminal end of PA0675; Ap^R^	This study
pUCPA0692	pUC18 carrying in SmaI-HindIII a 2.0-kb PCR fragment containing the *P. aeruginosa* PAO1 PA0692 gene, Ap^R^	This study
pUCPA0697	pUC18 carrying in EcoRI-HindIII a 848-bp PCR fragment containing the *P. aeruginosa* PAO1 PA0697 gene, Ap^R^	This study
pGST-0690	pRP270 carrying in BamHI-EcoRI a 653-bp PCR fragment containing the *P. aeruginosa* PAO1 C-terminal region of the PA0690 gene fused to GST, Ap^R^	This study
pGST-0692	pRP270 carrying the 567-bp NcoI-EcoRI insert from pUCPA0692 containing an internal fragment of the PA0692 gene fused to GST, Ap^R^	This study
pGST-0697	pRP270 carrying the 426-bp PstI-ApoI insert from pUCPA0697 containing an internal fragment of the PA0697 gene fused to GST, Ap^R^	This study
pMP0691b	PA0691 promoter fragment cloned upstream of *lacZ* gene in pMP220; Tc^R^	This study
pMP0691bKm	pMP0691b carrying in BglII, and in the opposite direction to the *lacZ* gene, a 1.2-kb BamHI fragment of pBSL99 containing a Km cassette; Km^R^, Tc^R^	This study
pMMB674HA	pMMB67EH carrying in EcoRI/HindIII a 0.8-kb PCR fragment of *vreA* (PA0674) with a C-terminal HA tag; Amp^R^	This study

a Ap^R^, Cm^R^, Km^R^, and Tc^R^, resistance to ampicillin, chloramphenicol, kanamycin, and tetracycline, respectively.

Bacterial strains were routinely grown in liquid Luria-Bertani (LB) medium [55] at 37°C on a rotary shaker operated at 200 revolutions per min. When required, antibiotics were used at the following final concentrations (µg mL^−1^): ampicillin (Ap), 100; chloramphenicol (Cm), 30; kanamycin (Km), 25 for *E. coli* and 200 for *P. aeruginosa*; piperacillin (Pip), 25; tetracycline (Tc), 10 for *E. coli* and 20 for *P. aeruginosa*.

### General molecular biology methods

Standard molecular biology techniques were used for DNA manipulations [55]. PCR amplifications and DNA sequencing were performed as described previously [19]. The sequences of the oligonucleotide primers used in this study are listed in Table S3 (Supporting Information). Transcriptional fusions to *lacZ* were made by cloning the promoter regions, amplified by PCR as EcoRI-XbaI or BglII-KpnI fragments, into the EcoRI-XbaI or BglII-KpnI sites of pMP220 [56]. The fusion constructs were confirmed by DNA sequencing, and transferred from *E. coli* DH5α to *P. aeruginosa* by triparental mating using the helper plasmid pRK600 as described before [57]. The influenza HA tag was cloned in the XbaI-HindIII sites of the pMUM3Rσno-stop plasmid (Table 2), which carries the *vreI* gene without stop codon, as an adapter that was the result of a primer dimer formed by the primers HAtagF-X and HAtagR-H (Table S3). This introduces the HA tag epitope YPYDVPDYAC* at the C-terminal end of the VreI protein. The resulting plasmid, in which the XbaI site is not restored, was designated pMUM3RσHA-tag. The HA tag was introduced at the end of the *vreA* gene, replacing the stop codon, by a PCR with primers PA0674F-E and EndPA06742. This introduces the HA tag epitope YPYDVPDYAC* at the C-terminal end of the VreA protein and an additional EcoRI and HindIII cloning site. These restriction enzymes were used to digest the PCR product after gel extraction and clone it in the brood host range vector pMMB67EH. The resulting plasmid was designated pMMB674HA.

### RNA isolation, cDNA probes synthesis and data analysis

*P. aeruginosa* cells bearing the plasmid pMMB67EH or pMUM3 were grown in quadruplicate in 300 mL Erlenmeyer flask with 30 mL LB and 25 µg/ml piperacillin at 37°C and 200 revolutions per min. In these conditions, the growth rate of the wild-type strain and the *vreI* overexpressing strain was similar (not shown), therefore cell density-dependent regulatory circuits are not affected. When the optical density at 600 nm reached 0.7–0.8, cultures were induced with 1 mM IPTG. After 45 min of incubation, a total of 50 ml of cells from two independent cultures were harvested by centrifugation at 4°C, and total RNA was isolated as described before [3]. RNA quantity was assessed by UV absorption at 260 nm in a ND-1000 Spectrophotometer (NanoDrop Technologies, USA). RNA quality was monitored by 1.5% (wt/v) agarose gel electrophoresis containing 2,2 M formaldehyde as denaturing agent. The cDNA probes were prepared according to the protocol supplied by the manufacturer (Affymetrix) as described before [3]. Target hybridization, washing, staining and scanning were performed by the Affymetrix Core Facility using a GeneChip® hybridization oven, a Fluidics station and MICROARRAY SUITE software (Affymetrix) at the Leiden Genome Technology Center (LGTC®) (Leiden, The Netherlands) as described previously [3]. Genes were considered differentially regulated if the relative change (*n*-fold) was >2.5 and the *P*-value was <0.05. Microarray data sets are available from the NCBI Geo Database under accession number GSE15697.

### RT-PCR analysis

RT-PCR analyses were performed by using the Titan One-Tube RT-PCR kit (Roche) in accordance with the manufacturer's recommendations. For each reaction, 1 µg of total RNA was used. The annealing temperature in each reaction was determined according to the composition of the primers included. DNA contamination of the RNA samples was ruled out by inactivation of the reverse transcriptase at 94°C for 4 min prior the RT-PCR reaction. The sequences of the primers used for the RT-PCR are listed in Table S4 (Supporting Information).

### Enzyme assays

ß-galactosidase activities in soluble cell extracts were determined using ONPG (Sigma-Aldrich) as described previously [19]. Each assay was run in duplicate at least three times and the data given are the average. The β-galactosidase activity is expressed in Miller Units.

### Purification of GST-tagged proteins and immunodetection

Overnight cultures of *E. coli* DH5α cells bearing the pRP270 (empty plasmid), or the pGST-0690, pGST-0692 and pGST-0697 plasmids (containing the indicated GST fusions) in LB liquid medium supplemented with ampicillin were subcultured 1∶10 in 500 ml of the same medium, grown until log-phase and incubated 3 h with 0.1 mM IPTG. The cells were harvested by centrifugation, resuspended in 10 ml of 1% (v/v) Triton X-100 in phosphate-buffered saline (PBS), and ultrasonically disrupted. The total bacterial lysate was centrifuged (12.000×g, 15 min, 4°C) and the supernatants loaded on Glutathione Sepharose 4B column (Pharmacia) equilibrated with 3 column volumes of PBS. GST-fused proteins were eluted with 10 mM glutathione in 50 mM Tris-HCl pH 8.0 in a fraction of 750 µl (3× times with 250 µl). Protein concentration was determined by the BCA protein assay (Pierce) using BSA as a standard.

Purified proteins were separated on a 12.5% (w/v) acrylamide SDS-PAGE gel and electrotransferred onto nitrocellulose and immunodetected with the serum of 25 different *P. aeruginosa* infected patients. The second antibody, horseradish peroxidase-conjugated goat anti-human, was visualized using 4-chloronaphtol/3,3-diaminobenzidine staining [55].

### SDS-PAGE and immunoblot

*P. aeruginosa* cells were grown in LB until late log phase and cultures were then centrifuged (10 min at 15,000×g). The cell pellets were solubilized in Laemmli sample buffer [58] and heated for 5 min at 95°C (total protein fraction). To separate cytosol and membrane fractions, cell pellets were first ultrasonically disrupted and centrifuged 5 min at 2,000×g to remove unbroken cells. The resulting supernatant was then centrifuged during 45–60 min at 12.000×g, 4°C. The pellet from this centrifugation step (membrane fraction) was solubilized in Laemmli buffer, and the proteins from the supernatant (cytosol fraction) were precipitated with 10% (w/v) trichloroacetic acid. Proteins from cell lysates, membrane and cytosol fractions were separated by SDS-PAGE containing 15% acrylamide. Proteins were electrotransferred onto nitrocellulose and immunodetected with a monoclonal antibody directed against the HA epitope. The second antibody, horseradish peroxidase-conjugated goat anti-mouse, was visualized using ECL detection (Pierce). Quantification was performed on a Fluor-S MultiImager (Bio-Rad) using Bio-Rad multianalyst software, version 1.0.2.

### Zebrafish embryos infection

Zebrafish embryos were collected from a laboratory-breeding colony kept at 24°C on a 12∶12 h light/dark rhythm as previously described [48]. Embryos were staged at 28 hours post-fertilization (hpf) dechorionated and anaesthetised in 0.02% buffered 3-aminobenzoic acid methyl ester (MS222, Sigma). Overnight cultures of *P. aeruginosa* cells bearing the pMMB67EH empty plasmid or the pMUM3 plasmid overexpressing *vreI* in LB liquid medium supplemented with piperacillin were subcultured 1∶50 in the same medium, grown until log-phase and incubated 1 h with 1 mM IPTG to induce *vreI* expression from the P*tac* promoter. Then, 2 ml of the log-phase bacteria was pelleted by centrifugation, washed twice with PBS and diluted in phenol red containing PBS (Sigma) at the desired bacterial density. Embryos were individually infected by microinjection (2 nl) of *P. aeruginosa* in the caudal vein near the blood island and the urogenital opening as previously described [48]. To determine the number of CFU microinjected in each set of embryos, bacteria were also microinjected in PBS and plated on LB-agar.

### Isolation of the mCherry DsRed variant

The mCherry variant of DsRed [59] was obtained as derivative of the pRSET-B plasmid (Invitrogen). In order to optimize for high gene expression in bacteria a new Shine/Dalgarno (SD) sequence was introduced upstream of the original ATG initiation codon. This was achieved by cloning an adapter that was the result of a primer dimer formed by the primers WBcherF (5′-GATCCAAGCTTGA**GGAGGA**-3′) and WBcherR (5′-GATC**TCCTCC**TCAAGCTTG-3′) in the BamHI site of the pRSET-B mCherry plasmid. This cloning introduced a HindIII site (underlined) and a SD sequence (GGAGGA) (shown in bold) in front of the mCherry gene. In order to centre the new SD on the -10 position from the ATG start codon, a 7 bp fragment between the SD and the ATG codon was then deleted. This was achieved by an inverse PCR reaction using DNA from this new plasmid as template and the primers Cherry-TomatoF (5′-CATGGTCAGCAAGGGCCAGG-3′) and WBcherR. The obtained PCR product was then re-ligated and transformed in *E. coli* DH5α cells. The resulting mCherry gene was then cloned as a HindIII-EcoRI fragment in the pBBR-PoprF plasmid, a derivative of the broad-host range pBBR1MCS-5 plasmid [60] (Table 2). This plasmid contains the promoter region of the *P. aeruginosa oprF* gene to maximize mCherry expression. This final construct, designated pBPF-mCherry, was introduced in *P. aeruginosa* by triparental mating [57].

### Whole mount immunnohistochemistry

For whole-mount immunohistochemistry, embryos were fixed in 4% (w/v) paraformaldehyde overnight at 4°C, rinsed in PBS and incubated in blocking solution for 1 hour (PBS+0.1% (v/v) Triton X-100+10% (v/v) normal goat serum). The primary antibody, a monoclonal anti-acetylated tubulin (Sigma, clone 6-11B-1) was diluted 1∶250 in PBS with 0.1% (v/v) Triton X-100 and 1% (v/v) normal goat serum, and incubated overnight at room temperature with slow agitation. After extensive washing with 0.1% (v/v) Triton X-100 in PBS, embryos were incubated overnight at room temperature in PBS with 0.1% (v/v) Triton X-100, 1% (v/v) normal goat serum and 1∶250 diluted secondary antibody, a goat anti-mouse conjugated to Alexa 480 (Invitrogen). After extensive washing in PBS with 0.1% (v/v) Triton X-100, embryos were transferred to Vectashield mounting medium (Vector Laboratories) and examined by microscopy.

### Microscopy analyses

Fixed or live zebrafish embryos, anaesthetised in 0.02% buffered MS222, were examined with a Leica MZ16FA stereomicroscope equipped with a DFC420C digital camera. Photographs were taken with the Leica Application Suite software (version 2.8.1 © Leica Microsystems). In addition, whole-mount immuno labeled zebrafish embryos were examined with a Leica DMIRE2 confocal microscope using the Leica confocal software (version 2.6.1 © Leica Microsystems).

## Supporting Information

Figure S1 Alignment of mature PA0674 with the N-terminal part of known *P. aeruginosa* TonB-dependent receptor proteins. Alignment of mature *P. aeruginosa* VreA (PA0674) with mature *P. aeruginosa* pyoverdine (FpvA), pyochelin (FptA), ferrioxamine B (FoxA), ferrichrome (FiuA) and mycobactin (FemA) receptor proteins. FpvA, FoxA, FiuA and FemA are involved in CSS and contain an N-terminal extension, whereas FptA, which is not part of a CSS system, does not have this extension. Asterisks below the sequences indicate positions at which identical or similar residues are presented at least in four of the sequences. Number of amino acid residues is indicated.
(0.01 MB PDF)

Figure S2 The predicted bilobal structure of PA0674 based on homology modeling. (A) Predicted domain architecture of VreA. The N-terminal domain (NTD) of VreA (shown in green) is preceded by a signal sequence (SS). The NTD and the C-terminal domain (CTD) (shown in blue) are separated by a short linker region. (B) The VreA/NTD (modeled residues 39–120) is classified as belonging to the Secretin/TonB N-terminus domain superfamily. Members of this superfamily have domains that are homologous to the N-terminal signaling domains of TonB-dependent outer membrane receptors (e.g. FpvA; PDB ID: 2O5P). The VreA/CTD (modeled residues 133–233) is expected to belong to the TolA/TonB C-terminal domain superfamily. The linker region separating the domains is shown in grey.
(0.07 MB PDF)

Figure S3 Localization of VreA in both wild-type cells and the *vreR* mutant. An HA-tagged version of the *vreA* gene was cloned in the broad-host range vector pMMB67EH under control of the *tac* promoter and subsequently introduced in mPAO1 wild-type strain and the *vreR* mutant (PA0676::ISphoA/hah). Cells were grown in LB without IPTG induction, disrupted sonication and both the soluble proteins and washed membrane fractions were isolated. Membrane proteins were loaded in five fold excess as compared to the soluble proteins. Proteins were separated on a 15% SDS-PAGE gels and the VreA-HA protein was visualized using antibodies directed against the HA tag. Molecular weight markers are shown on the left in kDa.
(0.19 MB PDF)

Figure S4 Schematic model for the *P. aeruginosa* PUMA3 CSS system. In this study we show that the PUMA3 CSS system is composed of three components, VreA, VreR and VreI. VreA is predicted to be composed of two domains, one of which will probably interact with the periplasmic domain of VreR, whereas the other domain might interact with (an) unknown outer membrane receptor(s). The unknown inducing signal is transmitted through VreR to the cytoplasm were it results in the activation of the alternative ECF sigma factor VreI. Activated VreI binds the RNA polymerase (RNAP) core enzyme and directs it two the VreI-dependent promoters. C, cytoplasm; CM, cytoplasmic membrane; OM, outer membrane; P, periplasm.
(0.16 MB PDF)

Figure S5 Association of PUMA3 with PUMA3-regulated genes in other bacteria. (A) Protein association network in STRING centered on the *P. aeruginosa* VreA (PA0674) protein. Two functional modules can be seen in the network, one showing the physical connectivity of *vreA* (*pigC*) with the other two PUMA3 components *vreI* (*pigD*) and *vreR* (*pigE*), and with some of the PUMA3-regulated genes (*i.e*. PA0690/ *tpsA*, PA0692/ *tpsB*, *exbB2*, *exbD2*, PA0696 and PA0697), and the other showing the functional connectivity of PUMA3 with components of other *P. aeruginosa* CSS systems (*i.e*. the sigma factor regulators PA0150, PA1911, PA3409 and PA4895, or the ECF sigma factor PA1912). (B) Schematic representation of the conserved PUMA3 gene neighborhood. Genes connected by lines are direct neighbors on the chromosome, and genes with the same color are orthologs across the various microorganisms.
(0.28 MB PDF)

Table S1 Genes of the VreI (PA0675) regulon activated in *P. aeruginosa* following 12 h of interaction with PNHAE epithelial cells (adapted from reference [20]).
(0.01 MB PDF)

Table S2 Genes of the VreI (PA0675) regulon upregulated in wild-type PAO1 exposed to epithelial cells versus grown in TSB (adapted from reference [21]).
(0.02 MB PDF)

Table S3 Oligonucleotide primers used in this work.
(0.08 MB PDF)

Table S4 Oligonucleotide primers used in the RT-PCR assays.
(0.03 MB PDF)
