# Supplementary material for: A Novel Extracytoplasmic Function (ECF) Sigma Factor Regulates Virulence in Pseudomonas aeruginosa
Source: PLoS Pathog. 2009 Sep 4;5(9):e1000572. doi: 10.1371/journal.ppat.1000572 (PMC2729926; doi:10.1371/journal.ppat.1000572)
Supplement: Figure S1 — Alignment of mature PA0674 with the N-terminal part of known P. aeruginosa TonB-dependent receptor proteins. Alignment of mature P. aeruginosa VreA (PA0674) with mature P. aeruginosa pyoverdine (FpvA), pyochelin (FptA), ferrioxamine B (FoxA), ferrichrome (FiuA) and mycobactin (FemA) receptor proteins. FpvA, FoxA, FiuA and FemA are involved in CSS and contain an N-terminal extension, whereas FptA, which is not part of a CSS system, does not have this extension. Asterisks below the sequences indicate positions at which identical or similar residues are presented at least in four of the sequences. Number of amino acid residues is indicated. (0.01 MB PDF) [file ppat.1000572.s001.pdf]

```
                      10        20        30        40        50
                       |         |         |         |         |
PA2398-FpvA   -------------QEVEFDIPPQALGSALQEFGRQADIQVLYRPEEVRNK
PA4221-FptA   --------------------------------------------------
PA2466-FoxA   -------AEAAAEQARPYAIPAGQLGDVLNRFAREAGITLSATPAQTGGY
PA0470-FiuA   -------APAEQASVRDYRIAAGPLAGTLNRIAAQAGLVLTLDPALAEGR
PA1910-FemA   ------AEAPAASSARSYDIAPGPLGRTLSAFASDNGVSLAFDPALTEGR
PA0674-VreA   AVPGDGGRPPLRLAVHAFDIPPQSLASALVTYSGVTGMAVLVDGELARGR
                               * *    **  *  **   ** *   *    **

                      60        70        80        90       100
                       |         |         |         |         |
PA2398-FpvA   RSSAIKGKLEPNQAITELLRGTGASVDFQGN-AITISVAEAADSSVDLGA
PA4221-FptA   -------------------------------------VADARKDGETELP
PA2466-FoxA   SSQGLRGSFTVQQGLARLLADTPLEAEDQGDGSFVLREAPAKDGDVLNMQ
PA0470-FiuA   SAHAVQGRFDAPGALREALKGSGLELVENAGGTYSLRKVPE---DTLSLQ
PA1910-FemA   RSAALRGRYAPVEALHRLLLGSGLELQQRSDGSYTLVPAAT--DGALELQ
PA0674-VreA   RSARLQGRFTASDALTRLLGGTGLMARYTSQEAFTLLPAQAVR-NAAPLP
               * ** ***    **  ** ****         ***  * *       *
```

Figure S1. Alignment of mature PA0674 with the N-terminal part of known *P. aeruginosa* TonB-dependent receptor proteins.

Alignment of mature *P. aeruginosa* VreA (PA0674) with mature *P. aeruginosa* pyoverdine (FpvA), pyochelin (FptA), ferrioxamine B (FoxA), ferrichrome (FiuA) and mycobactin (FemA) receptor proteins. FpvA, FoxA, FiuA and FemA are involved in CSS and contain an N-terminal extension, whereas FptA, which is not part of a CSS system, does not have this extension. Asterisks below the sequences indicate positions at which identical or similar residues are presented at least in four of the sequences. Number of amino acid residues is indicated.
