# Supplementary material for: A Novel Extracytoplasmic Function (ECF) Sigma Factor Regulates Virulence in Pseudomonas aeruginosa
Source: PLoS Pathog. 2009 Sep 4;5(9):e1000572. doi: 10.1371/journal.ppat.1000572 (PMC2729926; doi:10.1371/journal.ppat.1000572)
Supplement: Figure S2 — The predicted bilobal structure of PA0674 based on homology modeling. (A) Predicted domain architecture of VreA. The N-terminal domain (NTD) of VreA (shown in green) is preceded by a signal sequence (SS). The NTD and the C-terminal domain (CTD) (shown in blue) are separated by a short linker region. (B) The VreA/NTD (modeled residues 39–120) is classified as belonging to the Secretin/TonB N-terminus domain superfamily. Members of this superfamily have domains that are homologous to the N-terminal signaling domains of TonB-dependent outer membrane receptors (e.g. FpvA; PDB ID: 2O5P). The VreA/CTD (modeled residues 133–233) is expected to belong to the TolA/TonB C-terminal domain superfamily. The linker region separating the domains is shown in grey. (0.07 MB PDF) [file ppat.1000572.s002.pdf]

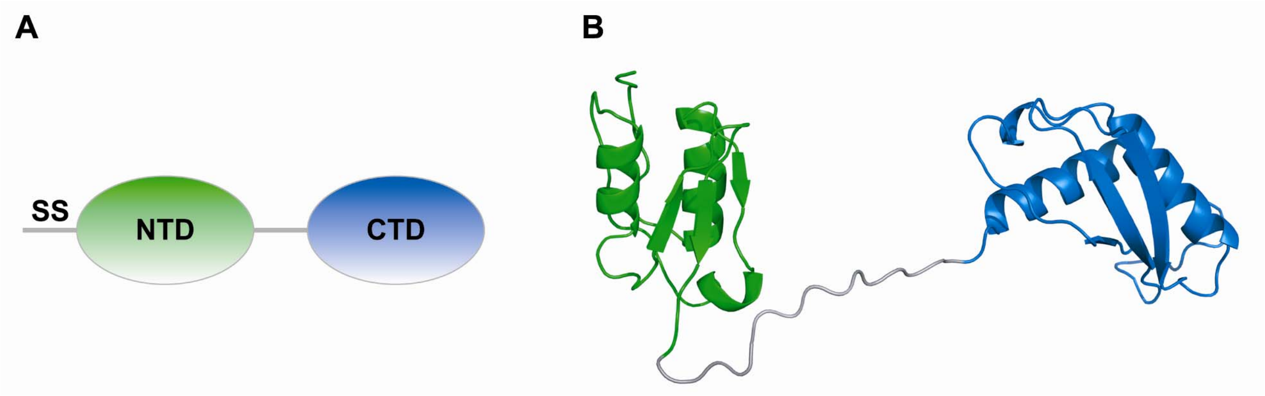

Figure S2. The predicted bilobal structure of PA0674 based on homology modeling.
(A) Predicted domain architecture of VreA. The N-terminal domain (NTD) of VreA (shown in green) is preceded by a signal sequence (SS). The NTD and the C-terminal domain (CTD) (shown in blue) are separated by a short linker region. (B) The VreA/NTD (modeled residues 39-120) is classified as belonging to the Secretin/TonB N-terminus domain superfamily. Members of this superfamily have domains that are homologous to the N-terminal signaling domains of TonB-dependent outer membrane receptors (e.g. FpvA; PDB ID: 2O5P). The VreA/CTD (modeled residues 133-233) is expected to belong to the TolA/TonB C-terminal domain superfamily. The linker region separating the domains is shown in grey.
