# Supplementary material for: A Novel Extracytoplasmic Function (ECF) Sigma Factor Regulates Virulence in Pseudomonas aeruginosa
Source: PLoS Pathog. 2009 Sep 4;5(9):e1000572. doi: 10.1371/journal.ppat.1000572 (PMC2729926; doi:10.1371/journal.ppat.1000572)
Supplement: Figure S3 — Localization of VreA in both wild-type cells and the vreR mutant. An HA-tagged version of the vreA gene was cloned in the broad-host range vector pMMB67EH under control of the tac promoter and subsequently introduced in mPAO1 wild-type strain and the vreR mutant (PA0676::ISphoA/hah). Cells were grown in LB without IPTG induction, disrupted sonication and both the soluble proteins and washed membrane fractions were isolated. Membrane proteins were loaded in five fold excess as compared to the soluble proteins. Proteins were separated on a 15% SDS-PAGE gels and the VreA-HA protein was visualized using antibodies directed against the HA tag. Molecular weight markers are shown on the left in kDa. (0.19 MB PDF) [file ppat.1000572.s003.pdf]

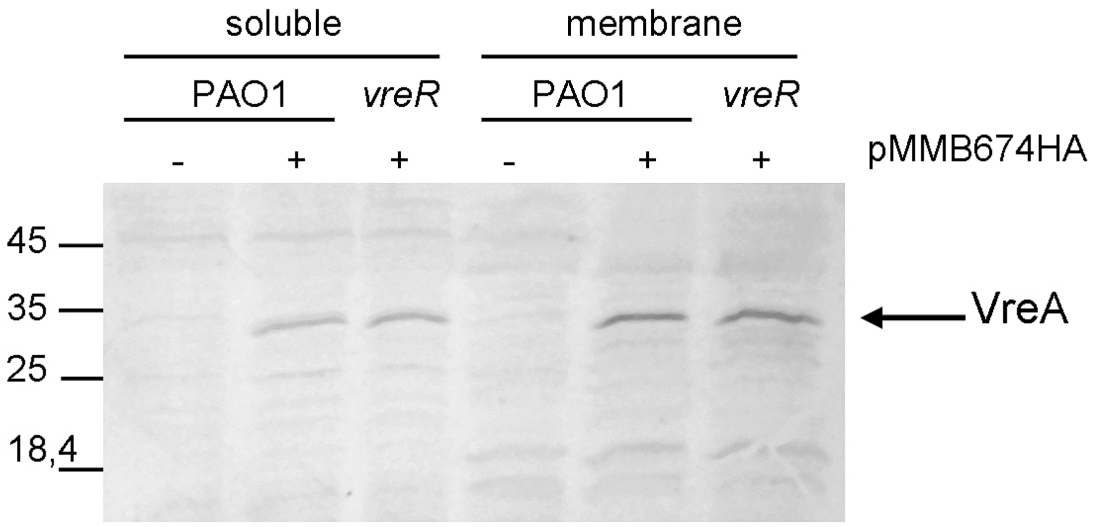

Figure S3. Localization of VreA in both wild-type cells and the *vreR* mutant.
An HA-tagged version of the *vreA* gene was cloned in the broad-host range vector pMMB67EH under control of the *tac* promoter and subsequently introduced in mPAO1 wild-type strain and the *vreR* mutant (PA0676::ISphoA/hah). Cells were grown in LB without IPTG induction, disrupted sonication and both the soluble proteins and washed membrane fractions were isolated. Membrane proteins were loaded in five fold excess as compared to the soluble proteins. Proteins were separated on a 15% SDS-PAGE gels and the VreA-HA protein was visualized using antibodies directed against the HA tag. Molecular weight markers are shown on the left in kDa.
