# Supplementary material for: A Novel Extracytoplasmic Function (ECF) Sigma Factor Regulates Virulence in Pseudomonas aeruginosa
Source: PLoS Pathog. 2009 Sep 4;5(9):e1000572. doi: 10.1371/journal.ppat.1000572 (PMC2729926; doi:10.1371/journal.ppat.1000572)
Supplement: Figure S4 — Schematic model for the P. aeruginosa PUMA3 CSS system. In this study we show that the PUMA3 CSS system is composed of three components, VreA, VreR and VreI. VreA is predicted to be composed of two domains, one of which will probably interact with the periplasmic domain of VreR, whereas the other domain might interact with (an) unknown outer membrane receptor(s). The unknown inducing signal is transmitted through VreR to the cytoplasm were it results in the activation of the alternative ECF sigma factor VreI. Activated VreI binds the RNA polymerase (RNAP) core enzyme and directs it two the VreI-dependent promoters. C, cytoplasm; CM, cytoplasmic membrane; OM, outer membrane; P, periplasm. (0.16 MB PDF) [file ppat.1000572.s004.pdf]

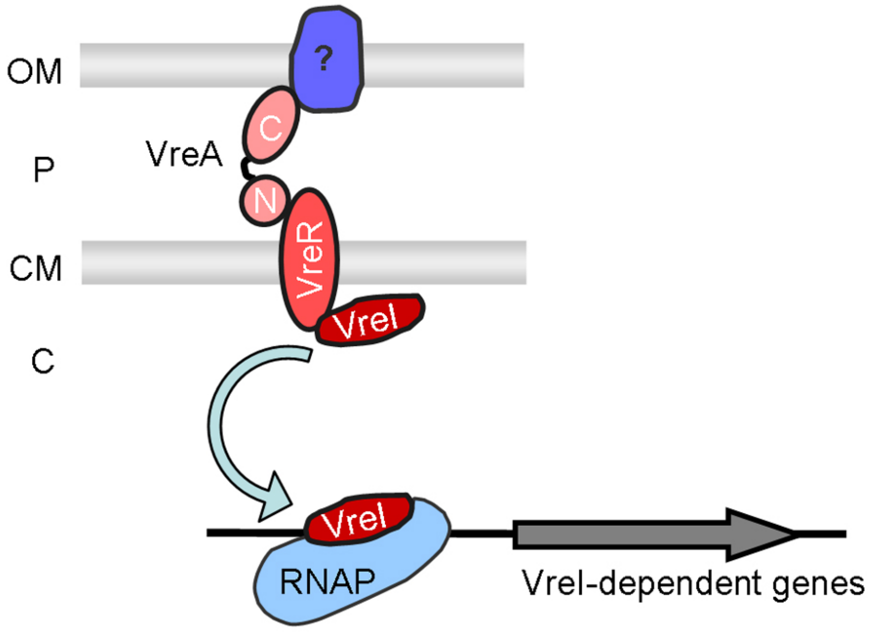

Figure S4. Schematic model for the *P. aeruginosa* PUMA3 CSS system.
In this study we show that the PUMA3 CSS system is composed of three components, VreA, VreR and VreI. VreA is predicted to be composed of two domains, one of which will probably interact with the periplasmic domain of VreR, whereas the other domain might interact with (an) unknown outer membrane receptor(s). The unknown inducing signal is transmitted through VreR to the cytoplasm were it results in the activation of the alternative ECF sigma factor VreI. Activated VreI binds the RNA polymerase (RNAP) core enzyme and directs it two the VreI-dependent promoters. C, cytoplasm; CM, cytoplasmic membrane; OM, outer membrane; P, periplasm
