# Supplementary material for: A Novel Extracytoplasmic Function (ECF) Sigma Factor Regulates Virulence in Pseudomonas aeruginosa
Source: PLoS Pathog. 2009 Sep 4;5(9):e1000572. doi: 10.1371/journal.ppat.1000572 (PMC2729926; doi:10.1371/journal.ppat.1000572)
Supplement: Figure S5 — Association of PUMA3 with PUMA3-regulated genes in other bacteria. (A) Protein association network in STRING centered on the P. aeruginosa VreA (PA0674) protein. Two functional modules can be seen in the network, one showing the physical connectivity of vreA (pigC) with the other two PUMA3 components vreI (pigD) and vreR (pigE), and with some of the PUMA3-regulated genes (i.e. PA0690/ tpsA, PA0692/ tpsB, exbB2, exbD2, PA0696 and PA0697), and the other showing the functional connectivity of PUMA3 with components of other P. aeruginosa CSS systems (i.e. the sigma factor regulators PA0150, PA1911, PA3409 and PA4895, or the ECF sigma factor PA1912). (B) Schematic representation of the conserved PUMA3 gene neighborhood. Genes connected by lines are direct neighbors on the chromosome, and genes with the same color are orthologs across the various microorganisms. (0.28 MB PDF) [file ppat.1000572.s005.pdf]

Figure S5

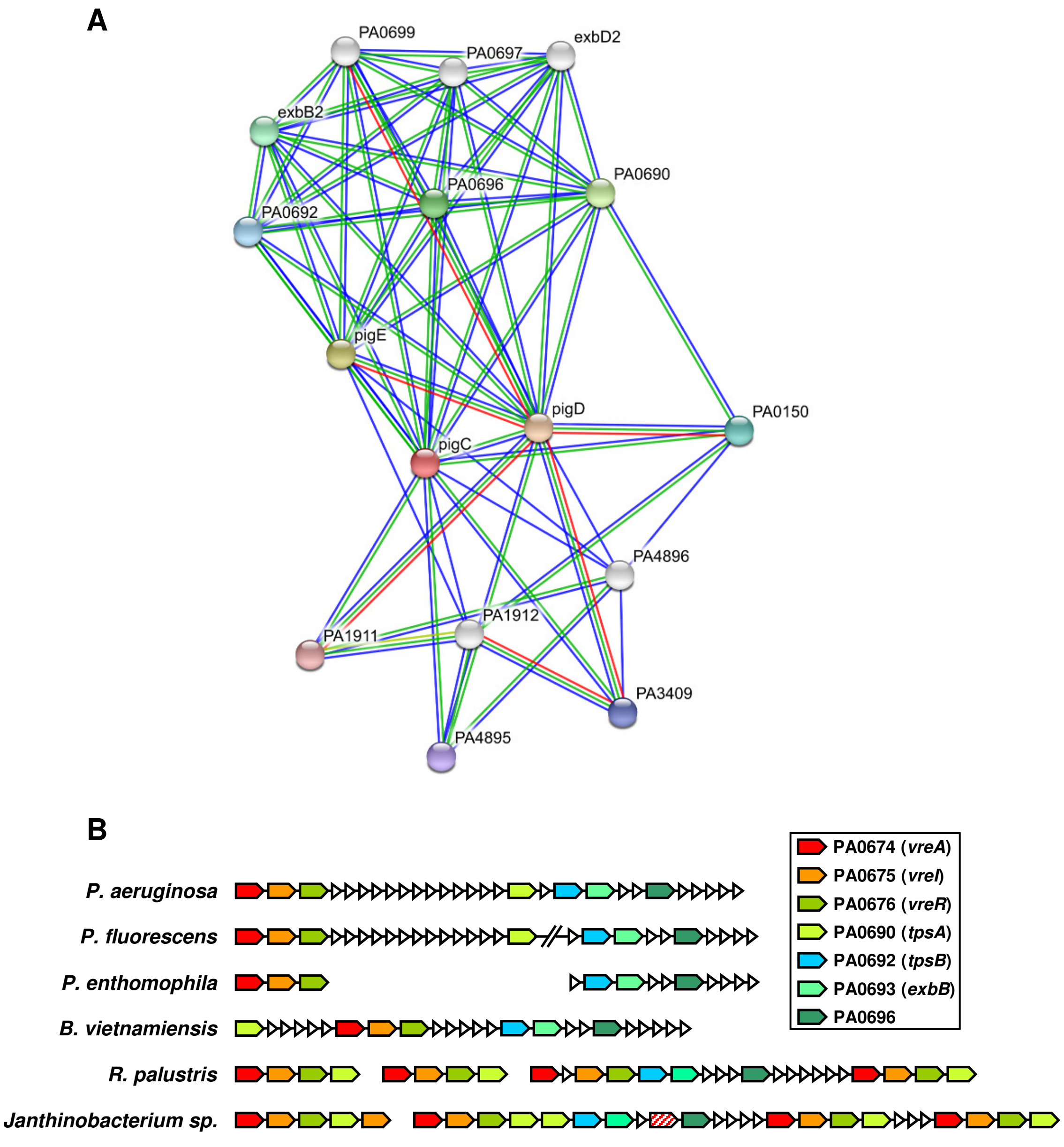

Figure S5. Association of PUMA3 with PUMA3-regulated genes in other bacteria.
(A) Protein association network in STRING centered on the *P. aeruginosa* VreA (PA0674) protein. Two functional modules can be seen in the network, one showing the physical connectivity of *vreA* (*pigC*) with the other two PUMA3 components *vreI* (*pigD*) and *vreR* (*pigE*), and with some of the PUMA3-regulated genes (*i.e.* PA0690/*tpsA*, PA0692/*tpsB*, *exbB2*, *exbD2*, PA0696 and PA0697), and the other showing the functional connectivity of PUMA3 with components of other *P. aeruginosa* CSS systems (*i. e.* the sigma factor regulators PA0150, PA1911, PA3409 and PA4895, or the ECF sigma factor PA1912). (B) Schematic representation of the conserved PUMA3 gene neighborhood. Genes connected by lines are direct neighbors on the chromosome, and genes with the same color are orthologs across the various microorganisms.
