# Supplementary material for: A Novel Extracytoplasmic Function (ECF) Sigma Factor Regulates Virulence in Pseudomonas aeruginosa
Source: PLoS Pathog. 2009 Sep 4;5(9):e1000572. doi: 10.1371/journal.ppat.1000572 (PMC2729926; doi:10.1371/journal.ppat.1000572)
Supplement: Table S1 — Genes of the VreI (PA0675) regulon activated in P. aeruginosa following 12 h of interaction with PNHAE epithelial cells (adapted from reference [20]). (0.01 MB PDF) [file ppat.1000572.s006.pdf]

TABLE S1. Genes of the VreI (PA0675) regulon activated in *P. aeruginosa* following 12 h of interaction with PNHAE epithelial cells (adapted from reference [20])

PA no.	Fold change[a]	Protein description and/or interspecies homology
PA0674/*pigC*	8.2	53% similar to ferripyoverdine receptor, PigC
PA0688	21.4	Hypothetical, 100% identical to PhoA
PA0691	19.0	Hypothetical, transposase
PA0693/*exbB2*	18.4	ExbB2, transport protein
PA0696	9.0	Hypothetical
PA0697	11.8	Hypothetical

[a]The magnitude of gene expression (fold change) was determined by comparing transcription in three replicates of PAO1 (control) with that in three replicates of PAO1 interacting with PNHAE cells after 12 h. Total RNA from the PNHAE cells alone (three replicates) was isolated, reverse transcribed into cDNA, and hybridized to the *Pseudomonas* GeneChip. The genes present from this experiment were subtracted from the analysis.
