# Supplementary material for: A Novel Extracytoplasmic Function (ECF) Sigma Factor Regulates Virulence in Pseudomonas aeruginosa
Source: PLoS Pathog. 2009 Sep 4;5(9):e1000572. doi: 10.1371/journal.ppat.1000572 (PMC2729926; doi:10.1371/journal.ppat.1000572)
Supplement: Table S2 — Genes of the VreI (PA0675) regulon upregulated in wild-type PAO1 exposed to epithelial cells versus grown in TSB (adapted from reference [21]). (0.02 MB PDF) [file ppat.1000572.s007.pdf]

Table S2. Genes of the VreI (PA0675) regulon upregulated in wild-type PAO1 exposed to epithelial cells versus grown in TSB (adapted from reference [21])

Gene*	Description*	Fold change†
PA0674/*pigC*	hypothetical protein (PigCDE CSS receptor)	99.8
PA0675/*pigD*	sigma-70 factor, ECF subfamily	9.0
PA0676/*pigE*	transmembrane sensor	9.5
PA0679/*hxcP*	hypothetical protein	8.3
PA0680/*hxcV*	HxcV putative pseudopilin (Type II secretion system)	24.5
PA0683/*hxcY*	probable type II secretion system protein	3.9
PA0688	low molecular weight alkaline phosphatase	3.8
PA0691	hypothetical protein (similar to transposase)	11.9
PA0692	outer membrane transporter of TPS pathway	8.9
PA0693/*exbB2*	transport protein ExbB2	6.9
PA0694/*exbD2*	transport protein ExbD	5.3
PA0696	hypothetical protein	60.0
PA0697	hypothetical protein	8.5
PA0698	hypothetical protein	13.1
PA0699	probable peptidyl-prolyl cis-trans isomerase, PpiC-type	6.3
PA2384	Probable Fur, Fe^{2+}/Zn^{2+} uptake regulation protein	27.7

*PA gene number, gene name and description are according to *Pseudomonas* Genome Project website (http://www.pseudomonas.com).
†Fold change in gene expression of wildtype PAO1 exposed to epithelial cells versus grown in TSB.
