# Supplementary material for: A Novel Extracytoplasmic Function (ECF) Sigma Factor Regulates Virulence in Pseudomonas aeruginosa
Source: PLoS Pathog. 2009 Sep 4;5(9):e1000572. doi: 10.1371/journal.ppat.1000572 (PMC2729926; doi:10.1371/journal.ppat.1000572)
Supplement: Table S3 — Oligonucleotide primers used in this work. (0.08 MB PDF) [file ppat.1000572.s008.pdf]

TABLE S3. Oligonucleotide primers used in this work

Amplified gene(s)	Plasmid[a]	Name	Sequence (5'→3')[b]
PA0675-PA0676	pUCMA3	Puma3F	CACTCGTCCGGGTACCTAT
		Puma3R	CTGAGCGAGATCCCATTGC
PA0674-PA0675	pMUM3Rσ	PA0674F-E	AATGAATTCCGGTGCATGGCGATTCTCCC
		PA0675R-B	TATGGATCCCCTGCTTATGCTTATGACGG
PA0674-PA0675(without stop codon)	pMUM3Rσno-stop	PA0674F-E	AATGAATTCCGGTGCATGGCGATTCTCCC
		PA0675R-X	AAATCTAGAGACGGTTTTCCGGCCCC
PA0674-PA0675-PA0676	pMMB-PUMA3	PA0674F-E	AATGAATTCCGGTGCATGGCGATTCTCCC
		PA0676R-B	AAAGGATCCGCACGCAGCAAAGTCCGC
PA0692	pUCPA0692	PA0692F-E	AAAGAATTCGGGGATCTCATGCAGATGCC
		PA0692R-H	AAAAAGCTTCAGGCCCAGGCAGATCAGCG
PA0697	pUCPA0697	PA0697F-E	AAAGAATTCAGCTCCGACGAGGTCTACG
		PA0697R-H	AAAAAGCTTAAAGGTGTTCGATCAGGGTG
PA0690 (C-terminal)	pGST-0690	PA0690F-B	TTTGGATCCCCTTCGGCGGCAGTATATTGG
		PA0690R-E	AAAGAATTCCAGGATCAGGTTGCCGC
PA0674 promoter	pMP0674	PR35E	AAAGAATTCATTCATAGGACAAAGCC
		PR33X	AAATCTAGAACCGAGCAACGACACTG
PA0691 promoter	pMP0691b	PR0691F2Bg	TAAAGATCTGATCGCCGCGCTGTTCCCCG
		PR0691RKp	TATGGTACCGATCAAGTTCCTGCATACCG
HA-tag epitope	pMUM3Rσ-HAtag	HAtagF-X	CTAGCCTACCCGTACGACGTGCCGGACTACGCGTGCTAA
		HAtagR-H	AGCTTTAGCACGCGTAGTCCGGCACGTCGTACGGGTAGG
HA-tag epitope	pMMB674HA	PA0674F-E	AATGAATTCCGGTGCATGGCGATTCTCCC
		EndPA0674.2	CTAAGCTTTAGCACGCGTAGTCCGGCACGTCGTACGGGTACCCTCCAACTCCCTCCGT

[a] The name of the plasmid containing the corresponding PCR product is indicated (see Table 2 for details)

[b] Restriction sites within the primers are underlined
