# Supplementary material for: A Novel Extracytoplasmic Function (ECF) Sigma Factor Regulates Virulence in Pseudomonas aeruginosa
Source: PLoS Pathog. 2009 Sep 4;5(9):e1000572. doi: 10.1371/journal.ppat.1000572 (PMC2729926; doi:10.1371/journal.ppat.1000572)
Supplement: Table S4 — Oligonucleotide primers used in the RT-PCR assays. (0.03 MB PDF) [file ppat.1000572.s009.pdf]

TABLE S4. Oligonucleotide primers used in the RT-PCR assays

Gene	Size	Name	Sequence (5'→3')
PA0691	198 bp	PA0691F1	TTGGCAGGGGGCGCTCAAGG
		PA0691R1	GGGAATCGCGTACAGCAGTTGC
PA0692	111 bp	RT-PA0692F	CCTGAACAACGACTACAGCG
		RT-PA0692R	GCGGTGAAATAGGTCAGCGA
PA0636	210 bp	PA0636F	CTGTCGCTTGCCAACGTGGG
		PA0636R	GAAGACCTGCGACTGGAGCG
